# Resolution of the *Hypoxylon fuscum* Complex (Hypoxylaceae, Xylariales) and Discovery and Biological Characterization of Two of Its Prominent Secondary Metabolites

**DOI:** 10.3390/jof7020131

**Published:** 2021-02-11

**Authors:** Christopher Lambert, Mohammad Javad Pourmoghaddam, Marjorie Cedeño-Sanchez, Frank Surup, Seyed Akbar Khodaparast, Irmgard Krisai-Greilhuber, Hermann Voglmayr, Theresia E. B. Stradal, Marc Stadler

**Affiliations:** 1 Helmholtz-Centre for Infection Research GmbH, Department of Microbial Drugs, Inhoffenstraße 7, 38124 Braunschweig, Germany; christopher.lambert@helmholtz-hzi.de (C.L.); javad.pormoghadam@gmail.com (M.J.P.); Marjorie.Cedeno@helmholtz-hzi.de (M.C.-S.); frank.surup@helmholtz-hzi.de (F.S.); 2 German Centre for Infection Research (DZIF), Partner Site Hannover-Braunschweig, 38124 Braunschweig, Germany; 3 Department of Plant Protection, Faculty of Agricultural Sciences, University of Guilan, Rasht 4199613776, Iran; blumeria2015@gmail.com; 4 Department of Botany and Biodiversity Research, University of Vienna, Rennweg 14, 1030 Wien, Austria; irmgard.greilhuber@univie.ac.at (I.K.-G.); hermann.voglmayr@univie.ac.at (H.V.); 5 Institute of Forest Entomology, Forest Pathology and Forest Protection, Department of Forest and Soil Sciences, BOKU-University of Natural Resources and Life Sciences, Franz Schwackhöfer Haus, Peter-Jordan-Straße 82/I, 1190 Vienna, Austria; 6 Helmholtz-Centre for Infection Research GmbH, Department for Cell Biology, Inhoffenstraße 7, 38124 Braunschweig, Germany; 7 Institute of Microbiology, Technische Universität Braunschweig, Spielmannstraße 7, 38106 Braunschweig, Germany

**Keywords:** analytical chemistry, Ascomycota, bioactivity screening chemotaxonomy, molecular phylogenetics, polyphasic taxonomy, two new species

## Abstract

*Hypoxylon,* a large, cosmopolitan genus of Ascomycota is in the focus of our current poly-thetic taxonomic studies, and served as an excellent source for bioactive secondary metabolites at the same time. The present work concerns a survey of the *Hypoxylon fuscum* species complex based on specimens from Iran and Europe by morphological studies and high performance liquid chromatography coupled to mass spectrometry and diode array detection (HPLC-MS-DAD). Apart from known chemotaxonomic markers like binaphthalene tetrol (BNT) and daldinin F, two unprece-dented molecules were detected and subsequently isolated to purity by semi preparative HPLC. Their structures were established by nuclear-magnetic resonance (NMR) spectroscopy as 3′-malonyl-daldinin F (**6**) and pseudofuscochalasin A (**4**). The new daldinin derivative **6** showed weak cytotoxicity towards mammalian cells but bactericidal activity. The new cytochalasin **4** was compared to cytochalasin C in an actin disruption assay using fluorescence microscopy of human osteo-sarcoma U2OS cells, revealing comparable activity towards F-actin but being irreversible compared to cytochalasin C. Concurrently, a multilocus molecular phylogeny based on ribosomal and proteinogenic nucleotide sequences of *Hypoxylon* species resulted in a well-supported clade for *H. fuscum* and its allies. From a comparison of morphological, chemotaxonomic and phylogenetic evidence, we introduce the new species *H. eurasiaticum* and *H. pseudofuscum*.

## 1. Introduction

The Hypoxylaceae are a family of the Xylariales (Sordariomycetes) that presently comprises 19 genera and approximately 400 taxa [1]. The family is characterized by the presence of stromatal pigments and a nodulisporium-like anamorph [2]. Most taxa in the family are saprobes, while some occur as endophytes and/or are associated with insect vectors [3]. The largest genus in the Hypoxylaceae is the type genus *Hypoxylon* with presently ca. 180 species. The generic concept of *Hypoxylon* was traditionally based on macromorphological features relating to the stromatal morphology until Ju and Rogers [4] restricted the genus to accommodate only those species that have a nodulisporium-like anamorph. Furthermore, they divided these anamorph stages into sub-types based on the complexity of the branching patterns of the conidiophores. Other genera whose species were traditionally placed in *Hypoxylon* (e.g., *Nemania*) are now accommodated in the Xylariaceae sensu stricto (see overview by Daranagama et al. [5]). Molecular studies have later validated this concept and also led to the segregation of further genera like *Annulohypoxylon* [6], *Hypomontagnella* [7], *Jackrogersella,* and *Pyrenopolyporus* [2] from the bulk of *Hypoxylon*. The current concept of the Hypoxylaceae is based on a multi gene genealogy but was recently even validated by a phylogenomic study based on 12 representatives using third generation genome sequencing techniques like Oxford nanopore and PACBIO [8]. However, both the multilocus phylogenetic tree and the phylogenomic reconstruction indicated that *Hypoxylon* is still polyphyletic and will need to be further segregated in the future. Until this can be accomplished, several complicated species complexes in the genus remain to be studied in-depth.

The *Hypoxylon fuscum* complex is one of them that needs additional comprehensive studies with polyphasic approaches. It is widely distributed in North temperate areas (Europe, North America) with occasional records from the tropics, the latter of which mostly came from higher altitudes [4]. The stromata are most frequently encountered on species of the Betulaceae but may grow on various other angiospermous host plants. A special case is *Hypoxylon porphyreum*, which is constantly associated with *Quercus* and was first reported by Granmo [9] from Norway, and later also found in France and (among the old herbarium specimens housed in New York Botanical Gardens) even in USA [10]. The latter study [10], which relied on HPLC profiling and morphological studies of several hundreds of herbarium specimens including many types, also confirmed that *H. macrosporum*, a species that is mostly known from *Salix* in the boreal-montane climates of the Northern hemisphere is related to *H. fuscum*. This was actually revealed by chemotaxonomic methodology in conjunction with morphological traits. In addition, the *H. fuscum* complex includes the (sub-)tropical *H. anthochroum*, the European *H. fuscoides* and various other potentially undescribed species that are thus far only known from old herbarium specimens [10,11].

The *Hypoxylon fuscum* complex is characterized by effused (on barkless wood) to effused-pulvinate (on bark), purple to red-brown stromata and olivaceous stromatal pigments in KOH. These major stromatal secondary metabolites of *H. fuscum* s. str. are binaphthalene tetrol (BNT; **1**) and azaphilones like daldinins C, E and F (**2**, **3**, **5**) [12]. The latter compounds are also present in *H. macrosporum* but are actually lacking in *H. porphyreum*, which contains similar, yet unidentified azaphilones that could so far not be isolated due to their instability and the scarcity of the material [10]. The conidiogeneous states of these species as well as of the variants that are for now accommodated in *H. fuscum* have a characteristic virgariella-like branching pattern that was first described by Petrini [13] for various isolates derived from stromata growing on *Alnus*, *Betula*, *Carpinus,* and *Corylus* and it does not seem to be possible to use this feature to further segregate them based on anamorphic traits. However, Petrini et al. [14] also studied the ascospore size ranges of numerous specimens from different host plants and found striking correlations. These data were not taken into account by Ju and Rogers [4], who reported a very large ascospore size range (8–20 µm × 4–8 µm) for the specimens they treated as *H. fuscum*, but notably, their broad concept had even included *H. porphyreum*. In fact, Stadler and Fournier [15] and Stadler et al. [16] had already pointed out that specimens referable to *H. fuscum* ss. Ju and Rogers [4] had a deviating chemotype from the “typical” variant of the species, which frequently occurs on *Corylus avellana* in Europe. The study by Stadler et al. [10] included the holotype of *Sphaeria fusca* from the 18th century and revealed that this material has exactly the same metabolite profile as recently collected material, i.e., BNT (**1**) and daldinins C (**2**), E (**3**) and F (**5**) were still detected by HPLC in the ancient stromata as major components. For this reason, Wendt et al. [2] have selected an epitype from *Corylus* in Germany originating from the previous work by Triebel et al. [17] that was cultured. The ex-epitype culture CBS 113049 can be used in the future to further stabilize the taxonomy of this species complex.

Iran (especially the north of Iran) includes subtropical regions and houses numerous species of Xylariales. Only few surveys on their diversity have so far been conducted in the country [18,19,20,21], and even the Iranian species of *Hypoxylon* are still poorly known and need further examination.

In the present study we have examined several specimens belonging to the *H. fuscum* complex from Iran and Europe using the above described combination of morphology, chemotaxonomy and molecular phylogeny. During the course of this work we also have isolated some new molecules that can serve as chemotaxonomic markers and report their physicochemical characteristics and biological activities. The present paper is dedicated to reporting these findings.

## 2. Materials and Methods

### 2.1. Sample Sources

Samples were collected from Iran and Europe. Iranian samples were collected from Guilan and Mazandaran provinces (Northern Iran) during 2015–2017. European samples were collected during an excursion of the European mycological congress in the Białowieża national Park (Poland), in the vicinity of Braunschweig, Germany and near Engelhartstetten in Lower-Austria. Parts of corticated branches and trunks bearing Hypoxylaceae stromata were transferred to the laboratory. Details of the specimens used for morphological investigations are listed in the Taxonomy section under the respective descriptions. Iranian specimens have been deposited in the fungarium of the Department of Plant Protection, Faculty of Agricultural Science, University of Guilan, Guilan, Iran (GUM). European specimens were deposited in the herbarium of the Staatl. Museum für Naturkunde, Karlsruhe, Germany (KR) and in the fungarium of the University of Vienna, Austria (WU). Living cultures were deposited in the culture collection MUCL (Louvain, Belgium) and DSMZ (Braunschweig, Germany). All fungal names used in this work and the corresponding taxonomy follow the recent overview on families of Sordariomycetes [1].

### 2.2. Morphological Characterisation

Morphological analyses of microscopic characters were carried out as described by Pourmoghaddam et al. [21]. Pigment colors were determined as described in the latter monograph, with colour codes following Rayner [22]. Macrophotographs were obtained with a Keyence VHX-6000 microscope. Light microscopy with Nomarski differential interference contrast (DIC) was done using a Zeiss Axio Imager.A1 compound microscope, equipped with a Zeiss Axiocam 506 colour digital camera. SEM of ascospores were recorded using a field-emission scanning electron microscope (FE-SEM Merlin, Zeiss, Germany), in a similar fashion as reported previously [23].

### 2.3. DNA Extraction, PCR and Sequencing

DNA extraction of fresh cultures and amplification of the ITS (nuc rDNA internal transcribed spacer region containing ITS1-5.8S-ITS2), LSU (5′ 1200 bp of the large subunit nuc 28S rDNA), *rpb2* (partial second largest subunit of the DNA-directed RNA polymerase II), and *tub2* (partial β-tubulin) loci were performed as described by [2]. DNA Sequences were generated by an in-house Sanger capillary sequencing solution on the HZI campus and processed with Geneious^®^ 7.1.9 (http://www.geneious.com) [2].

### 2.4. Molecular Phylogenetic Analyses

The newly generated sequences were aligned with selected sequences from [2] and a combined matrix of the four loci (ITS, LSU, *rpb2* and *tub2*) was produced for phylogenetic analyses, with four species (*Biscogniauxia nummularia*, *Graphostroma platystomum*, *Xylaria arbuscula,* and *Xylaria hypoxylon*) added as the outgroup. The GenBank accession numbers of sequences are listed in Table 1. Sequences were aligned with the server version of MAFFT (http://mafft.cbrc.jp/alignment/server/), checked and refined using BioEdit v7.2.6 [24,25].

Maximum Parsimony (MP) analyses were performed with PAUP v4.0a165 [26]. All molecular characters were unordered and given equal weight; analyses were performed with gaps treated as missing data; the COLLAPSE command was set to MINBRLEN. MP analysis of the combined multilocus matrix was done using 1000 replicates of heuristic search with random addition of sequences and subsequent TBR branch swapping (MULTREES option in effect, steepest descent option not in effect). Bootstrap analyses with 1000 replicates were performed in the same way but using 10 rounds of random sequence addition and subsequent branch swapping during each bootstrap replicate.

Maximum Likelihood (ML) analyses were performed with RAxML [27] as implemented in raxmlGUI 1.3 [28], using the ML + rapid bootstrap setting and the GTRGAMMA substitution model with 1000 bootstrap replicates. The matrix was partitioned for the different gene regions. Bootstrap values ≤70% are considered low, between 70 and 90% intermediate and ≥90% high in the discussion of the data that follows further below.

### 2.5. HPLC Profiling

Stromata from *Hypoxylon* specimens were extracted using acetone as described previously [23] and subsequently analysed by high-performance liquid chromatography coupled to a diode array and electrospray mass spectrometric detection system (HPLC/DAD-ESIMS) with instrument settings as described recently [21]. Resulting UV/Vis and mass spectrometric data were compared with internal databases, comprising standards of known Hypoxylaceae and literature data [10,11,12].

### 2.6. Extraction and Isolation of Compounds ***4***, ***5***, and ***6***

For a chemical study, 337 mg of stromata derived from the specimen 987 GUM was extracted five times with acetone as stated previously by sonication and subsequent filtration [23] with a yield of 85.8 mg extract. The crude extract was subjected to a Strata X-33 µm reversed-phase (RP) column to remove fatty acids and debris. The constituents of the pre-cleaned extract (47.6 mg) were further separated using a C18-Gemini 10 µm; 250 × 21 mm column in a PLC 2250 preparative HPLC system (Gilson, Middleton, WI, USA) with gradient settings as follows: isocratic conditions (equilibration) for 6.25 min, 60% H_2_O, 40% acetonitrile (ACN), 10 mL/min; gradient from 40% ACN to 55%, 45 min, 20 mL/min; gradient from 55% ACN to 100% ACN for 5 min, 20 mL/min; isocratic conditions for 10 min at 100% ACN, 20 mL/min. Fractions were collected for every 10 mL of solvent volume. Fractions at Retention time (RT) = 19−20.5 (I) and 35−37.5 min (II) were collected and dried in vacuo.

In a second attempt, 209 mg of stromata were extracted as stated previously to give rise to 64 mg of crude extract from strain 987 GUM and pre-cleaned using a Strata-X 33 µm column to remove fatty acids, debris and to achieve a pre-fractionation by using different solvent mixtures. A solvent mix of 80% H_2_O and 20% ACN resulted in a 23.87 mg fraction (**III**). Stromatal extracts of specimens 217H and 303H (134 mg and 199 mg dry mass, resulting in 39 mg and 27 mg crude extract in total) were pooled and fractionated following a similar strategy, however, with a solvent mixture of 59.5% H_2_O, 39.5% ACN with 1% formic acid (FA), resulting in a 48.7 mg fraction (IV).

Fraction **I** was purified by NP-TLC (pre-coated TLC plates Silgur-25, Macherey-Nagel, Düren, Germany) with 100 mL mobile phase comprised of 80% dichloromethane (DCM) and 20% acetone as solvent. TLC material was scraped and adsorbed compound eluted by acetone + 0.1% formic acid, which resulted in 0.3 mg of pure compound (**4**).

Fraction **II** was purified by NP-HPLC (Gilson, Middleton, WI, USA; GX-271 liquid handler, two pumps; 305 and 306, DAD wavelengths set to 210, 254 and 391 nm) with the mobile phase comprised of 50% isopropanol (**A**) and 50% of a solvent mixture containing 60% EtAc, 20% benzene, 20% n-heptane and 1% formic acid (**B**) to 100% B in 50 min. Fractions from RT = 9.3−10.7 min were combined to give rise to 2.1 mg of compound **5**. An Orbit 100 5µm diol column (MZ-Analysentechnik GmbH, Mainz, Germany) was used as stationary phase.

Fraction **III** was purified by NP-TLC with 100 mL mobile phase comprised of 40% EtAc, 30% benzene, 30% n-heptane and 1% FA and subsequent elution similar to fraction **I**, which lead to 0.46 mg of pure compound (**5**).

Fraction **IV** was purified by NP-TLC with 100 mL mobile phase comprised of 4% EtAc, 30% benzene, 30% n-heptane and 1% FA and subsequent elution similar to fraction **I**, which lead to 3.27 mg of pure compound (**6**).

### 2.7. Spectral Data

#### 2.7.1. Pseudofuscochalasin A (**4**) Figures S1–S6

Colorless oil. [α]^25^_D_ = +166 (*c* 0.3, AcN); ^1^H NMR (700 MHz, CHCl_3_-*d*): *δ* ppm 7.33 (t, *J* = 7.4 Hz, 3′–H/5′–H), 7.25 (d, *J* = 7.4 Hz, 4′–H), 7.17 (d, *J* = 7.4 Hz, 2′–H/6′–H), 6.88 (d, *J* = 15.5 Hz, 20–H), 6.45 (d, *J* = 15.5 Hz, 19–H), 5.93 (ddd, *J* = 15.6, 10.2, 0.9 Hz, 13–H), 5.61 (br s, 2–NH), 5.20 (ddd, *J* = 15.6, 10.9, 5.0 Hz, 14–H), 4.10 (br s, 7–OH), 4.05 (d, *J* = 9.8 Hz, 7–H), 3.55 (br s, 4–H), 3.41 (td, *J* = 7.6, 1.3 Hz, 3–Ha), 2.73 (dqd, *J* = 10.9, 6.8, 1.4 Hz, 16–H), 2.603 (m, 10–H_2_), 2.598 (m, 15–H_a_), 2.082 (m, 8–H), 2.078 (m, 15–H_b_), 1.67 (br s, 12–H_3_), 1.64 (s, 23–H_3_), 1.44 (s, 11–H_3_), 1.24 (d, *J* = 6.8 Hz, 22–H_3_), ^13^C NMR (175 MHz, CHCl_3_-*d*): *δ* ppm 209.7 (C, C–17), 197.6 (C, C–21), 173.6 (C, C–1), 145.0 (CH, C–19), 137.1 (C, C–1′), 135.0 (CH, C–14), 133.6 (CH, C–20), 131.8 (C, C–6), 130.2 (CH, C–13), 129.2 (2 × CH, C–2′/C–6′), 128.8 (2 × CH, C–3′/C–5′), 127.0 (CH, C–4′), 126.2 (C, C–5), 78.8 (C, C–18), 69.3 (CH, C–7), 63.2 (C, C–9), 59.0 (CH, C–3), 53.6 (CH, C–8), 47.3 (CH, C–4), 43.0 (CH, C–16), 42.9 (CH_2_, C–10), 38.3 (CH_2_, C–15), 23.5 (CH_3_, C–23), 19.8 (CH_3_, C–22), 17.2 (CH_3_, C–11), 14.1 (CH_3_, C–12), ESIMS *m/z* 464.29 ([M + H]^+^, 462.34 ([M − H]^-^, HRESIMS *m/z* 464.2430 ([M + H]^+^, calcd for C_28_H_34_NO_5_ 464.2431), 486.2250 ([M + Na]^+^, calcd for C_28_H_33_NO_5_Na 486.2251).

#### 2.7.2. Daldinin F (**5**) Figures S7–S11

^1^H NMR (500 MHz, acetone-*d*_6_) *δ* ppm 7.73 (d, *J* = 1.4 Hz, 1–H), 7.30 (d, *J* = 15.6 Hz, 3′′–H), 6.15 (d, *J* = 1.4 Hz, 5–H), 6.09 (br q, *J* = 7.0 Hz, 5′′–H), 5.86 (s, 4–H), 5.77 (d, *J* = 15.6 Hz, 2′′–H), 4.57 (d, *J* = 6.0 Hz, 3′–OH), 4.01 (m, 3′–H), 3.92 (dqd, *J* = 11.5, 6.2, 2.4 Hz, 6′–H), 2.15 (m, 4–H_a_), 2.05 (m, 2–H_3_), 1.88 (m, 4–H_b_), 1.812 (m, 5′–H_a_), 1.811 (d, *J* = 7.0 Hz, 6′’–H3), 1.77 (br s, 7′′–H3), 1.52 (m, 5′–H_b_), 1.45 (s, 7-Me), 1.10 (d, *J* = 6.2 Hz, 7′–H), ^13^C NMR (125 MHz, acetone-*d*_6_): *δ* ppm 194.5 (C, C–6), 192.5 (C, C–8), 169.8 (C, C–1′), 166.2 (C, C–1′’), 155.8 (CH, C–1), 151.8 (CH, C–3′′), 144.0 (C, C–4a), 138.7 (CH, C–5′’), 134.7 (C, C–4′’), 122.8 (CH, C–5), 115.0 (CH, C–2′’), 111.7 (C, C–8a), 103.9 (C, C–3), 86.0 (C, C–7), 69.8 (CH, C–6′), 67.2 (CH, C–4), 62.8 (CH, C–3′), 26.9 (CH_2_, C–4′), 26.6 (CH_2_, C–5′), 22.8 (CH_3_, 7Me), 21.7 (CH_3_, C–7′), 20.0 (CH_3_, C–2′), 14.7 (CH_3_, C–6′′), 11.8 (CH_3_, C–7′’); HRESIMS *m/z* 461.1806 ([M + H]^+^, calcd for C_24_H_29_O_9_ 461.1806), 483.1625 ([M + Na]^+^, calcd for C_24_H_28_O_9_Na 483.1625).

#### 2.7.3. 3′-Malonyl-daldinin F (**6**) Figures S12–S17

Yellow oil. [α]^25^_D_ = +18.0 (*c* 1.0, AcN). ^1^H NMR (700 MHz, acetone-*d*_6_) *δ* ppm 7.76 (d, *J* = 1.4 Hz, 1–H), 7.31 (d, *J* = 15.6 Hz, 3′′–H), 6.13 (d, *J* = 1.4 Hz, 5–H), 6.09 (br q, *J* = 7.1 Hz, 5′′–H), 5.84 (br s, 4–H), 5.73 (d, *J* = 15.6 Hz, 2′′–H), 5.11 (t, *J* = 2.8 Hz, 3′–H), 4.03 (dqd, *J* = 10.8, 6.2, 2.6 Hz, 6′–H), 3.48 (d, *J* = 15.9 Hz, 3′–′′–H_a_), 3.37 (d, *J* = 15.9 Hz, 3′–′′–Hb), 2.19 (m, 4–H_a_), 2.05 (m, 2–H_3_), 2.02 (m, 4–H_b_), 1.81 (d, *J* = 7.0 Hz, 6′′–H_3_), 1.76 (br s, 7′′–H_3_), 1.68 (m, 5′–H_a_), 1.64 (m, 5′–H_b_), 1.46 (s, 7-Me), 1.14 (d, *J* = 6.2 Hz, 7′–H), ^13^C NMR (125 MHz, acetone-*d*_6_): *δ* ppm 194.3 (C, C–6), 192.2 (C, C–8), 169.9 (C, C–1′), 167.9 (C, C–3′′′), 166.2 (C, C–1′′′), 166.1 (C, C–1′′), 155.1 (CH, C–1), 152.5 (CH, C–3′′), 143.0 (C, C–4a), 139.1 (CH, C–5′′), 134.7 (C, C–4′′), 123.0 (CH, C–5), 114.2 (CH, C–2′′), 111.8 (C, C–8a), 102.0 (C, C–3), 86.0 (C, C–7), 70.1 (CH, C–6′), 66.6 (CH, C–4), 65.7 (CH, C–3′), 41.8 (CH_2_, C–2′′′), 26.9 (CH_2_, C–5′), 24.0 (CH_2_, C–4′), 22.6 (CH_3_, 7Me), 21.5 (CH_3_, C–7′), 20.0 (CH_3_, C–2′), 14.7 (CH_3_, C–6′′), 11.8 (CH_3_, C–7′′); HRESIMS *m*/*z* 547.1811 ([M + H]^+^, calcd for C_27_H_31_O_12_ 547.1810), 569.1629 ([M + Na]^+^, calcd for C_27_H_30_O_12_Na 569.1629).

### 2.8. Antimicrobial Activities and Cytotoxicity

Evaluated substances were solved in a concentration of 1 mg/mL in methanol and tested against a panel of microorganisms (*Acinetobacter baumanii*, *Escherichia coli*, *Bacillus subtilis*, *Mycobacterium smegmatis*, *Staphylococcus aureus*, *Pseudomonas aeruginosa*, *Chromobacter violaceum*) and fungi (*Schizosaccharomyces pombe*, *Pichia anomala*, *Candida albicans*, *Mucor hiemalis*) in a serial dilution assay [45]. Cytotoxicity was evaluated via a MTT-assay against two cell lines (L929 mouse fibroblasts, KB 3.1 endocervical carcinoma) as described previously [46].

### 2.9. Actin Disruption Assay

The actin disrupting potential of the newly described cytochalasin structurally related to cytochalasin C was investigated in the adherent mammalian osteosarcoma (U2OS, ATCC HTB-96) cell line by fluorescence microscopy as described [47]. Briefly, for the assay exponentially growing cells were seeded on fibronectin coated cover slips and allowed to spread overnight. On the next day, cells were treated with 5 and 1 µg/mL of the new compound dissolved in full medium for one hour. To probe reversibility, one set of treated cells was washed and incubated in fresh medium lacking compound for one hour prior to fixation. DMSO served as a negative control. Cells were washed and fixed with 4% para-formaldehyde for 20 min. Fixed cells were permeabilized using 0.1% Triton X-100 (Hercules, CA, USA) at room temperature for 1 min. Cytochalasin C is commercially available (Cayman chemical, MI, USA) and was used as a standard to for comparison with the novel cytochalasin due to its related structure. The actin cytoskeleton was stained with fluorescently-labelled phalloidin (ATTO-594, ATTO-Tec, Siegen, Germany) and the nucleus was stained with DAPI, contained in the mounting medium Pro-long Diamond Antifade (Invitrogen, Carlsbad, CA, USA). Pictures were recorded using an inverted microscope (Axio Vert 135 TV, Zeiss, Jena, Germany) equipped with a Coolsnap 4k camera (Photometrics, Tuscon, AZ, USA) operated by Metamorph software package (molecularDevices, San Jose, CA, USA) and processed by Image J (NIH, Bethesda, MD, USA).

## 3. Results

### 3.1. Molecular Phylogeny

**Figure 1:**
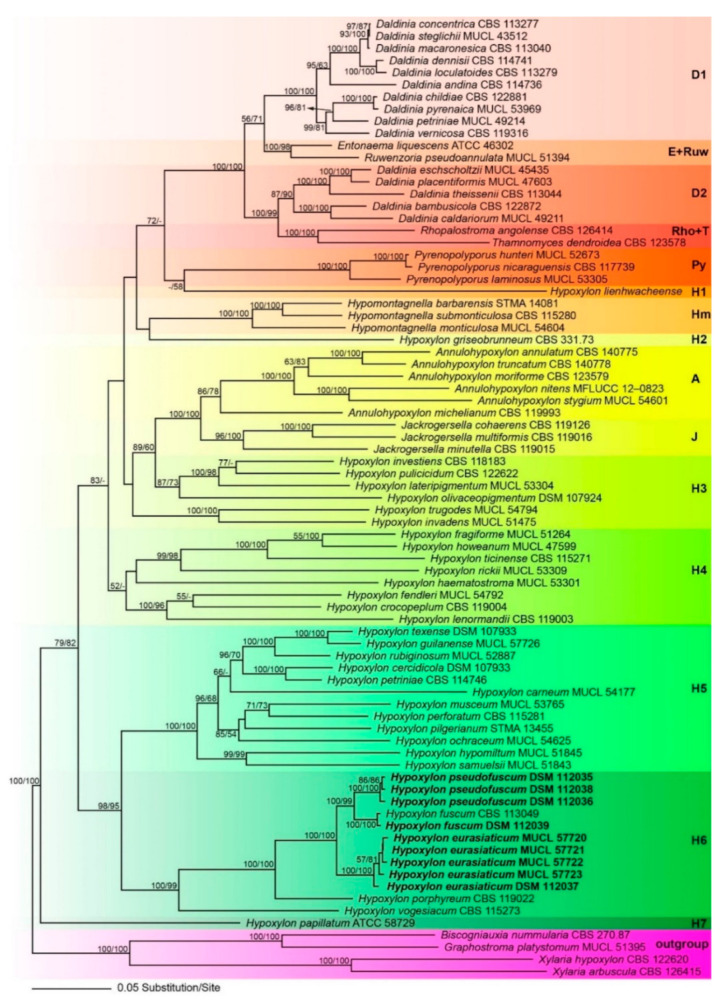
Phylogram of the best ML trees (lnL = −72650.213864) revealed by RAxML from an analysis of the combined ITS–LSU–*rpb2*–*tub2* matrix of selected Xylariales. Strains in bold were sequenced in the current study. ML and MP bootstrap support above 50% are given at the first and second positions, respectively, above or below the branches. Different background colours have been applied to highlight the major clades.

Of the 4256 nucleotide characters of the combined matrix, 1637 are parsimony informative (310 of ITS, 162 of LSU, 490 of *rpb2* and 675 of *tub2*). Figure 1 shows a phylogram of the best ML tree (lnL = −72,650.213864) obtained by RAxML. Maximum parsimony analyses revealed eight MP tree comprising 16,256 steps (data not shown). All major groups and deeper, highly supported nodes were consistent between the ML and MP analyses, but topologies of deeper unsupported nodes differed in the MP tree; as these differences are not relevant within the context of our new species, they are not further considered here. The phylogenies reveal a paraphyly of *Hypoxylon*, with the genera *Annulohypoxylon*, *Daldinia*, *Entonaema*, *Jackrogersella*, *Hypomontagnella*, *Pyrenopolyporus*, *Rhopalostroma*, *Ruwenzoria,* and *Thamnomyces* embedded within the former. 

All of the latter genera appeared monophyletic except for *Daldinia* (Figure 1). All of our new species described below are contained within the highly supported *Hypoxylon* clade H6. The new species (*Hypoxylon eurasiaticum*) is the sister group of *H. pseudofuscum* and *H. fuscum sensu stricto* (Figure 1). Sequences of *H. pseudofuscum* clustered together with *Hypoxylon fuscum* with 100% BS support. The sequences of the collection of *Hypoxylon fuscum* (DSM 112039) are almost identical to those of the ex-epitype culture and they clustered together with maximum support. The remaining clades are in accordance with previous results [2,7].

### 3.2. Taxonomic Part

***Hypoxylon eurasiaticum*** Pourmoghaddam, Krisai-Greilhuber & Khodap., sp. nov.

**Figure 3:**
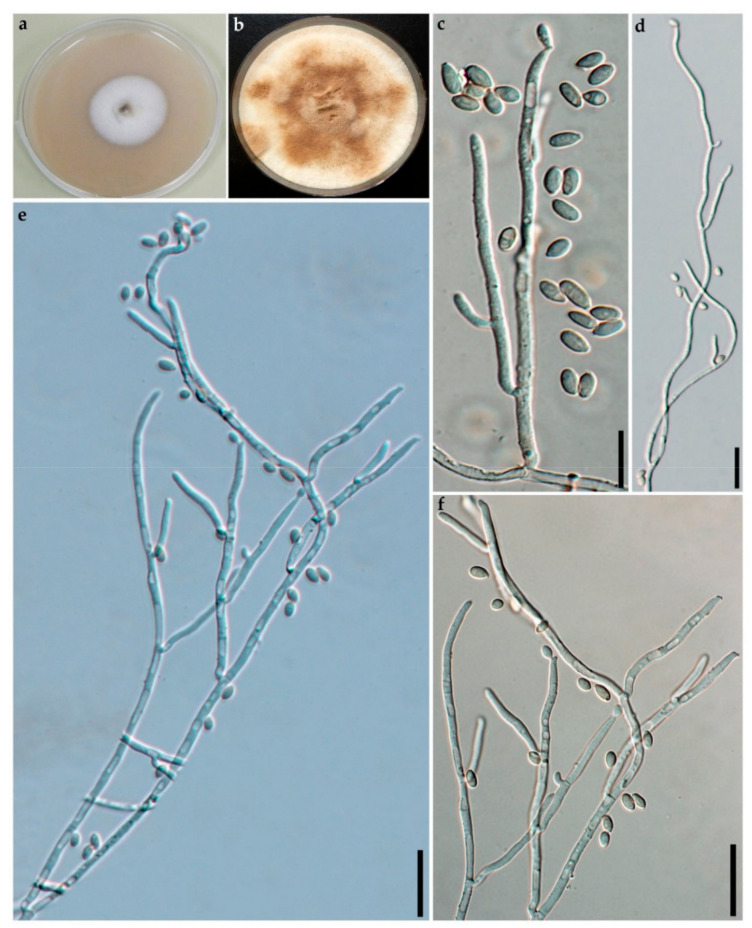
Culture and anamorphic structures of *Hypoxylon eurasiaticum* (GUM 1597) on OA. (**a,b**) surface of colony after 1 and 8 weeks of incubation (respectively left to right); (**c**–**f**) general view of anamorph structure with virgariella-like branching patterns, conidiogenous cells, immature and mature conidia. Scales bars set as (**c**–**f**) 20 µm.

**MycoBank** No: 838247, Figure 2 and Figure 3

**Etymology**. Eurasiaticum, for its occurrence in both, Europe and Asia.

**Holotype** (designated here) Iran, Guilan Province, Shaft County, Babarekab forest, 37°00′26.88′′ N, 49°20′22.95′′ E, 289 m elev., on fallen branch of *Quercus castaneifolia*, 15 September 2016, leg. M.J. Pourmoghaddam (GUM 1597; ex-holotype culture preserved in metabolically inactive state, MUCL 57720).

**Teleomorph**. Stromata superficial, hemispherical, pulvinate to effused-pulvinate, up to 12 cm long × 0.2–2 cm wide, with inconspicuous to slightly conspicuous perithecial mounds, surface Vinaceaous (57) or Dark Vinaceaous (82), Brown Vinaceous (84); dull orange to orange-brown granules beneath the surface and dark dull granules between the perithecia, with Amber (47) to Honey (64), Isabelline (65), Olivaceous (48) or Hazel (88) KOH-extractable pigments. Perithecia obovoid to spherical, 0.15–0.4 mm high × 0.1 mm–0.3 mm wide. Ostioles umbilicate, inconspicuous. Asci with amyloid, discoid apical apparatus, 0.5–1.5 µm high × 2.5–3.5 µm wide, stipe up to 60 µm, and spore-bearing portion 70–90 × 7–10 µm. Ascospores smooth, unicellular, brown to dark brown, ellipsoid, inequilateral with narrowly rounded ends, 9–12.5 × 4–6 µm, with more sigmoid to less straight germ slit spore-length on convex side; perispore dehiscent in 10% KOH, conspicuous coil-like ornamentation in SEM; epispore smooth.

**Cultures and anamorph**. Colonies on OA covering a 9 cm Petri dish in 2 w, at first white, becoming Straw (46) from outwards, cottony; finally, attaining Umber (9) or Ochraceous (44). Conidiogenous structure branching virgariella-like as defined by Ju and Rogers [4], (Figure 3c–f). Conidiophores hyaline, smooth to finely roughened. Conidiogenous cells hyaline, smooth to finely roughened, 15–23 × 2–3 µm. Conidia hyaline, smooth to ellipsoid, 4–6 × 2–4 µm.

**Secondary metabolites**. BNT, daldinin F, 3′malonyl-daldinin F, unknown compounds related to naphthalene secondary metabolite family.

**Other specimens examined**. Iran, Guilan Province, Langaroud County, Liseroud forest, 37°7′44′′ N, 50°8′41′′ E, 28 m elev., on fallen branch of *Quercus castaneifolia*, 10 September 2017, leg. M.J. Pourmoghaddam (GUM 1598; culture MUCL 57721); Guilan Province, Siahkal County, Lonak Waterfall, 37°00′31.12′′ N, 49°51′52.69′′ E, 500 m elev., on fallen branch of *Quercus castaneifolia*, 10 Nov 2016, leg. M.J. Pourmoghaddam (GUM 1600; culture MUCL 57722); Guilan Province, Fouman County, Masouleh forest, 37°09′23.83′′ N, 48°59′58.12′′ E, 863 m elev., on fallen branch of *Quercus castaneifolia*, 23 July 2015, leg. M.J. Pourmoghaddam (GUM 988; culture MUCL 57723), Poland, Podlaskie Voivodeship, Białowieża National Park, on branch of cf. *Betula*, 20 September 2019, leg. C. Lambert (KR-M-0005886, culture DSM 112039).

**Notes**. This taxon differs in having smaller ascospores than the two other taxa in the *Hypoxylon fuscum* complex (see phylogenetic analyses (Figure 1). In addition, morphological and chemotaxonomic data (see Table 2 and Table 3) supported our hypothesis. The lack of malonyl-daldinin F in *Hypoxylon fuscum* provided an additional argument to regard *Hypoxylon eurasiaticum* as a separate taxon in this complicated species complex. The apparent host preference for *Quercus castaneifolia* in Iran is also striking, since the other records of similar *Hypoxylon* species from *Quercus* (*H. porphyreum*, as well as the type specimens of *H. subchlorinum* and *H. commutatum* subsp. *holwayanum*, whose status is still unclear) previously studied [10] showed yet different morphological characters and also deviating HPLC profiles. However, the material from Poland definitely was not collected from *Quercus*, hence further studies on the host range of the new taxon should be carried out in the future based on these data.

***Hypoxylon pseudofuscum*** Pourmoghaddam, Khodap. & Krisai-Greilhuber, sp. nov.

MycoBank No: 838248, Figure 4

**Etymology**. Pseudofuscum, referring to the morphological similarity to *H. fuscum*.

**Holotype** (designated here) Germany, Rhineland-Palatinate, Bad Dürkheim, Lake “Isenachweiher”, on fallen branch of *Alnus glutinosa*, 7 June 2020, leg. Barbara and M. Stadler (STMA 18264, KR-M-0005879), ex-type culture deposited in metabolically inactive state in DSM 112038.

**Teleomorph**. Stromata superficial, pulvinate to effused-pulvinate, up to 6 cm long × 1–3 cm wide, with inconspicuous to slightly conspicuous perithecial mounds, surface Rust (39), Brick (59), Vinaceaous (57) or Dark Vinaceaous (82), Brown Vinaceous (84); dull orange to orange-brown granules beneath the surface and dark dull granules between the perithecia, with Amber (47), Isabelline (65), Olivaceous (48) or Hazel (88) KOH-extractable pigments. Perithecia spherical to obovoid, 0.19–0.36 high × 0.12–0.28 mm wide. Ostioles umbilicate, inconspicuous. Asci with amyloid, discoid apical apparatus, 0.5–1.5 µm high× 2–3.5 µm wide, stipe up to 55 µm, and spore-bearing portion 65–85 × 6–10 µm. Ascospores smooth, unicellular, brown to dark brown, ellipsoid, inequilateral with narrowly rounded ends, 11–16 × 4.5–7.3 µm, with sigmoid to less frequently straight germ slit spore-length on convex side; perispore dehiscent in 10% KOH; epispore smooth.

**Cultures**. Colonies on OA covering a 9 cm Petri dish in 2 weeks, at first white, cottony, becoming Pale Luteous (46) from outwards with concentric zones; finally, attaining Amber (47). Anamorph not observed.

**Secondary metabolites**. BNT, daldinin F, 3′malonyl daldinin F, pseudofuscochalasin A, one unknown compound with a conspicuous UV and two un-known compounds related to the naphthalene secondary metabolite family.

**Specimens examined**. Germany, Lower Saxony, Allerbüttel near Ilkerbruch, on *Salix* sp., 17 June 2020, leg. H. Andersson (KR-M-0005876; culture DSM 12036); Germany, Lower Saxony, Braunschweig, Viehmoor near Leiferde, on *Salix* sp., 17 June 2020, leg. H. Andersson (KR-M-0005877, culture DSM 112035 ); France, Pyrénées Atlantiques, Auterrive, Ile du Gave d’Oloron, on wood of *Alnus glutinosa*, 30 May 2004, leg. J. Fournier and M. Stadler (STMA 04048); Iran, Guilan Province, Talesh County, Gisoom forest, 37°39′41′′ N, 49°00′31′′ E, 11 m elev., on fallen branch of *Alnus* sp., 2 September 2015, leg. S. Raei (GUM 987).

**Notes**. This taxon is phylogenetically close to *Hypoxylon fuscum* (Figure 1). The phylogenetic analyses have good support to distinguish these two sister group. In addition, we found BNT, 3′-malonyl-daldinin F, and the new pseudofuscochalasin A as stromatal metabolites. It also differs from *Hypoxylon eurasiaticum* in having larger ascospores (see Table 2).

### 3.3. HPLC Profiling

Among the studied specimens of the *H. fuscum* complex, five were derived from Iran (GUM 987, GUM 988, GUM 1597, GUM 1598, GUM 1600), one from Poland KR-M-0005886), three from Germany (KR-M-0005877, KR-M-0005876 and KR-M-0005879), and one from Austria (WU 43621). Host substrate, origin and identified compounds by comparison with an internal database are shown in Table 3 and briefly summarized further below.

The major constituents were identified as compounds **1** and **5** together with two unidentified compounds (**UC 2** and **UC 3**, see supporting information for HRMS data) which share the typical crown-shape UV/vis pattern with BNT (1), pointing towards structural features shared with the naphthalene secondary metabolite family. Specimen growing on *Alnus* sp. were found to contain a member of the cytochalasin family and another unidentified compound with a conspicuous UV absorption pattern (**UC 1**). Compound **UC 1** was only present in the specimens not derived from *Corylus*. These results corroborated the molecular phylogenetic and morphological data that we concurrently obtained.

### 3.4. Structure Elucidation (Figure 5 and Figure 6)

The molecular formula of **4** was indicated as C_28_H_33_NO_5_ based on its [M + H]^+^ and [M + Na]^+^ peaks at m/z 464.2430 and 486.2250 in the HRESIMS spectrum, respectively, implying 13 degrees of unsaturation. ^1^H and HSQC NMR spectra revealed the presence of four methyls, two methylenes, and seven olefinic (two with dual intensity) as well as five aliphatic methines. In addition, the ^13^C spectrum specified two ketones, a carboxylic carbon, and five further carbons devoid of bound protons. HMBC correlations connected the ^1^H,^1H^ COSY and TOCSY spin systems to a cytochalasin skeleton. The closest known structural relative of 4 is cytochalasin C (**7**), which formally constitutes its 21-deacetyl-didehydro derivative.

Compound **4** shares the stereochemistry with **7**, which was confirmed by ROESY data. ROESY correlations between 13–H and 20–H on the α face as well as between 14–H and 19–H on the β face of the molecule supported the characteristic conformation described for the eleven-membered ring system, whereas the common 3*S*,4*R*,7*S* configuration can be assumed by analogous chemical shifts for the core structure as well as the biogenesis from l-Phe [48]. ROESY correlations between 23–H_3_ and 16–H as well as between 23–H_3_ and 19–H, located above the molecular main plain, endorse the upwards orientation of 23–H_3_ and thus an 18*R* configuration. We propose the trivial name pseudofuscochalasin for compound 4, whose systematic IUPAC name is (7S,13E,16S,18R,19E)-16,18-dimethyl-7,18-dihydroxy-10-phenyl [11]cytochalasa-5,13,19-triene 1,17,21-trione [13].

The molecular formulae of **5** and **6** were determined as C_24_H_28_O_9_ and C_27_H_30_O_12_ by their molecular ion clusters at *m*/*z* 461.1806 and 547.1811 in their HRESIMS spectra. The main metabolite **5** was identified as daldinin F by its ^1^H and ^13^C NMR data. Its derivative **6**, which differed from **5** by the formal addition of a C_3_H_2_O_3_ fragment, exhibited very similar NMR data. Key difference in ^1^H and HSQC spectra were the presence of an additional methylene group and the low-field shift of oxymethine CH_2_–3′. HMBC correlations from 2′′′–H_2_ to C–1′’’ and C–3′′′ identified a malonyl moiety, which was connected to C–3′ due to the HMBC correlation of 3′–H to C–1′′′. Consequently, **6** was established as 3′-malonyl-daldinin F. The positive optical rotation of both **5** and **6** indicated a common 7*S* absolute configuration for the daldinins C–F [12,49,50].

### 3.5. Antibacterial and Cytotoxic Activities

The biological activities compounds **5** and **6** against microorganisms and mammalian cells were evaluated against a panel of bacteria (MIC) and cell lines (L929, fibroblasts; KB 3.1, endocervical adenocarcinoma cells). Neither compound showed activity in the selected concentration range, except for weak cytotoxicity of **6**. (IC_50_ of 35 µg/mL against L929 murine fibroblasts and 17 µg/mL against Hela KB 3.1 cervix carcinoma cells), respectively.

### 3.6. Actin Disruption Assay (Figure 7)

**Figure 7:**
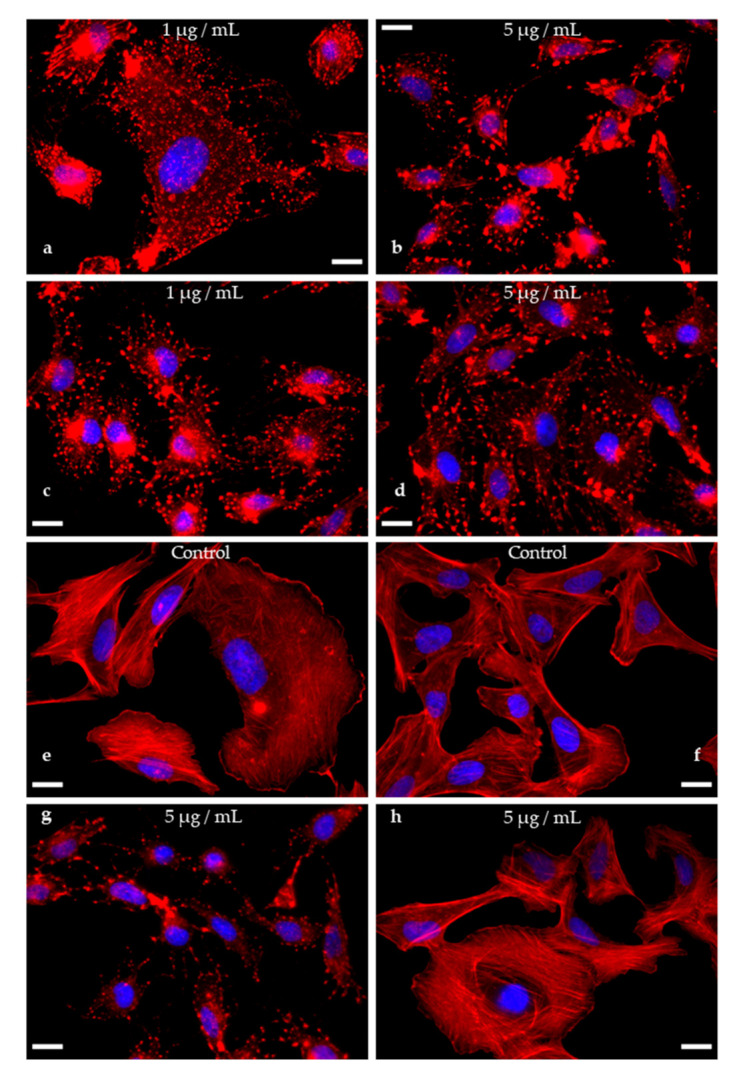
Overlay images of U2OS cells treated with compounds **4** ((**a**): 1 µg/mL; (**b**): 5 µg/mL; (**g**): 5 µg/mL, wash-out), 7 ((**c**): 1 µg/mL; (**d**): 5 µg/mL; (**h**): 5 µg/mL, wash-out) or vehicle control (DMSO, (**e**): 5 µl/mL; (**f**): wash-out control), PFA-fixed and stained for their actin cytoskeleton (phalloidin-ATTO594, red) and nuclei (DAPI, blue). Knot-like structures and aggregates of f-actin in treated cells demonstrate the disruptive effect of compounds **4** and **7** in comparison to the intact actin cytoskeleton depicted in the vehicle control (**e**). Note that the effect of **4** is not reversible, as a wash-out (**f**) with a recovery time of one hour did not lead to a regeneration of the f-actin network (compare (**b**,**g**)), while the effect conveyed by compound **7** was fully revertible (compare (**d**) and (**h**)). Scales bars set as 20 µm.

The new cytochalasin and its acetylated derivative were tested for its actin disrupting potential in a recently described actin disruption assay and a functional wash-out experiment with a one-hour of recovery time. With both, high and low concentrations, the typical consequences of an intoxication with cytochalasans arise as visible by f-actin aggregated knot-like structures (Figure 7b–d) as well as a reduction of stress-fiber-like structures in low-dose application of 4, where the degradation of the actin cytoskeleton was less pronounced (1 µg/mL; see Figure 7a). This reflects the well-known interference of cytochalasans with the fast-growing (barbed end) of f-actin filaments [51]. Interestingly, the effect of **4** is not functionally reversible as opposed to its acetylated derivative **7**, pointing towards the loss of the enone function at C-21 as a key trait for the reversibility of the actin cytoskeleton degradative capabilities. It will be interesting to further embark on this pattern and verify this feat in differently organized cytochalasan core structures such as chaetoglobosins and other cytochalasans.

## 4. Discussion

In this work, we characterized some specimens of the *H. fuscum* complex by a polyphasic approach using chemotaxonomic and multilocus sequencing data for the first time. This species complex had previously been recognized by the meticulous work of Petrini et al. [14], who had found correlations between the host plants from which the stromata were collected and the ascospore size range, but could not use the latter character to segregate species owing to the fact that the spore sizes were overlapping while the anamorphic structures from various mycelial cultures they made from specimens inhabiting different hosts were rather similar. The type specimen of *Sphaeria fusca* Pers., which is deposited in the fungarium of Leiden (L) represents the commonly encountered member of this species complex that is very frequently found on *Corylus avellana*. An epitype with matching characteristics was selected and sequenced as prerequisite to stabilize the taxonomy of the *H. fuscum* complex. While the broad concept of *H. fuscum* was upheld by Ju and Rogers [4], Mühlbauer et al. [52] provided first evidence on the existence of several chemotypes that could be useful for species segregation. Quang et al. [12] found that stromata of *H. fuscum* from *Corylus* contain daldinin C, which had previously been isolated from a *Daldinia* sp. and also described two congeners named daldinins E and F. Further HPLC profiling studies on *H. fuscum* specimen occurring on different hosts based on a large number of specimens revealed the existence of two different chemotypes [10,16]. Specimens from *Corylus* contains BNT, daldinal A, daldinins C, E and F, while a second chemotype mostly represented by specimens from *Alnus* and *Salix* were devoid of daldinal A and contained unidentified compounds. The identity of two of these metabolites was established in the current study as 3′ malonyl daldinin F (**6**) and the novel pseudofuscochalasin A (**4**). We have detected further yet unidentified compounds in the crude extracts but their isolation and structure elucidation was not possible, owing to the small amounts of stromatal material available.

The current work also provided a resolution of the *H. fuscum* species complex by molecular phylogeny for the first time. An earlier study based on ITS sequences using several vouchers of *H. fuscum* s. lat. showed no delimitation of *H. fuscum* across different host-species in a molecular phylogenetic inference [17]. However, the ITS sequences are of questionable utility in the Xylariales because of intraspecific and even intragenomic polymorphism on the one hand and high redundancies on the other hand [53,54]. Our current molecular phylogenetic analysis was congruent with published data, showing a paraphyly of *Hypoxylon*, which was not solved by the addition of the newly generated sequences. However, three clearly distinguished clades with high bootstrap support were formed by *H. fuscum* s. str. and its allies. Taking together morphological, chemical and phylogenetic data we introduce two new species. The previously reported specimens of the “*Alnus*/*Salix* chemotype” will have to be assigned to either *H. eurasiaticum* and *H. pseudofuscum* based on repetitive studies of their HPLC profiles (best using the standardized HPLC-MS system that was employed in the present study) and morphology. It remains possible that additional members of this species complex will be recognized as new taxa in the near future. This will definitely afford the availability of fresh material that can be cultured and subjected to a multi locus phylogeny.

The new chemotaxonomic marker metabolites were also subjected to a preliminary analysis of their biological effects, but due to the limited amounts available, no extensive activity spectra could be recorded. The difference in the activities of daldinin F, which was found inactive in our study, to previously reported data reported by Quang et al. [55], who had reported significant antibacterial effects for the compound, may be due to the use of different assay protocol. The new daldinin derivative **6** was also devoid of antimicrobial effects but showed only weak cytotoxicity against L929 murine fibroblasts and KB 3.1 cervix-cancer cells. The other new compound **4** belongs to the cytochalasins, which are well-known actin cytoskeleton disrupting agents with a huge structural diversity [56] and many different subtypes with varying biological effects have been reported. However, a clear-cut structure activity relationship is not yet available for this compound class and only few systematic studies were undertaken.

Recently we have established certain correlations between the chemical structures and the corresponding biological effects, such as the importance of an enone moiety for the general reversibility of the f-actin collapsing effect [47]. This was further corroborated by the comparison of four and seven in the present study. However, this adds only a small puzzle frame towards the many differently organized cytochalasin core structures, for which this pattern has to be verified.

## 5. Conclusions and Outlook

The present study has contributed further to the establishment of correlations between biological and chemical diversity of the fungal genus *Hypoxylon* and constitutes the first attempt to segregate a rather complicated species complex by using polyphasic taxonomy methodology and in particular the correlation of chemotaxonomy and a multi-locus genealogy has proven useful for this task. In the future additional specimens of the *H. fuscum* complex from other host plants and geographic areas should be included. It may also be of great interest to study the lichenicolous and endophytic strains that were assigned to “*H. fuscum*” (s. lat.) in the literature e.g., [57,58]. Unfortunately, the authors of these papers have apparently not deposited the cultures in public domain collections and they might not be accessible for phylogenetic studies. This is unfortunately the case for many other xylarialean endophytes that were reported to produce interesting secondary metabolites, and the reported taxonomy of the producer strains has frequently been inaccurate [59,60].

## Figures and Tables

**Figure 2:**
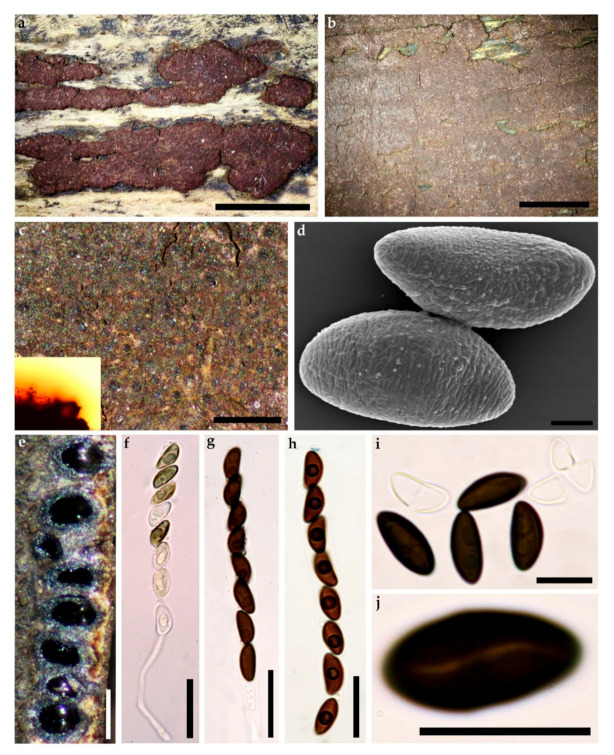
*Hypoxylon eurasiaticum* (Holotype GUM 1597). (**a**) stromatal habit; (**b**,**c**) close-up view of stromatal surface, with stromatal pigments in 10% KOH; (**d**) ascospore under SEM; (**e**) stroma in section showing perithecia and ostioles; (**f**) immature ascus in water; (**g**) mature ascus in water; (**h**) ascus in Melzer’s reagent; (**i**) ascospores in 10% KOH with dehiscent perispore; (**j**) ascospore in water, with sigmoid germ-slit. Scales bars set as (**a**,**b**) 5 mm; (**c**) 0.5 mm; (**d**) 2 µm; (**e**) 0.5 mm; (**f**–**h**) 20 µm; (**i**,**j**) 10 µm.

**Figure 4:**
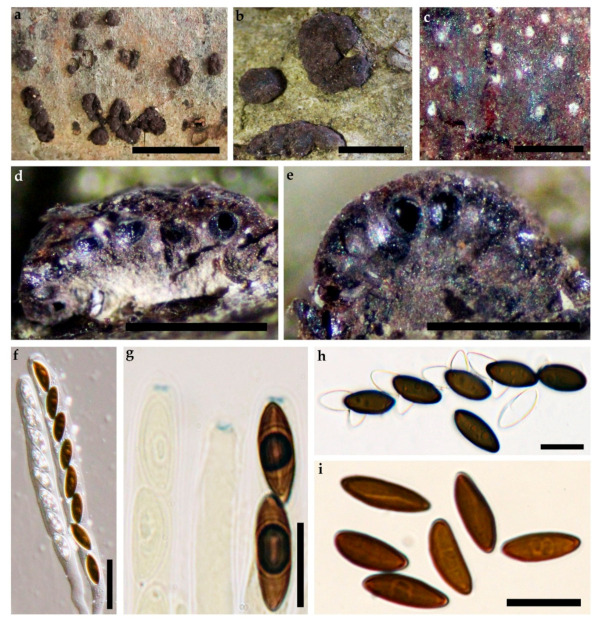
*Hypoxylon pseudofuscum* (Holotype). (**a**,**b**) stromatal habit; (**c**) close-up view of stromatal surface; (**d**,**e**) stroma in section showing perithecia and ostioles; (**f**) mature and immature ascus in water; (**g**) mature and immature asci tips in Melzer’s reagent; (**h**) ascospores in 10% KOH with dehiscent perispore; (**i**) ascospores in water, with sigmoid germ-slit. Scales bars set as (**a**) 1 cm; (**b**) 2.5 mm; (**c**–**e**) 1mm; (**f**) 20 µm; (**g**–**i**) 10 µm.

**Figure 5:**
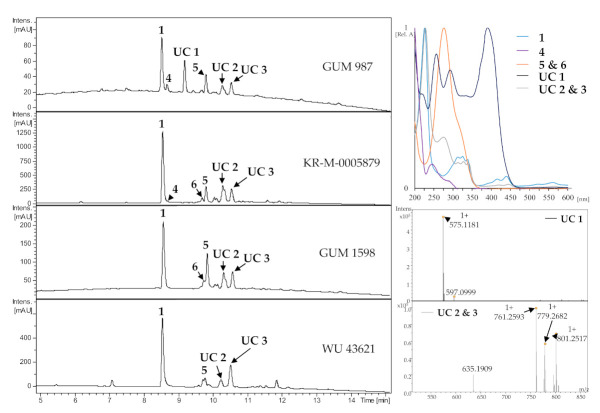
Representative HPLC chromatograms (UV, 210 nm) of stromatal acetone extracts from *H. fuscum* (WU 43621), *H. eurasiaticum* (GUM 1598) and *H. pseudofuscum* (GUM 987 and KR-M-0005879/holotype). UV-Vis chromatograms from 200 to 600 nm and HRESIMS data of unknown compounds of designated peaks are given together with their normalized absorption. UC: Unknown compounds.

**Figure 6:**
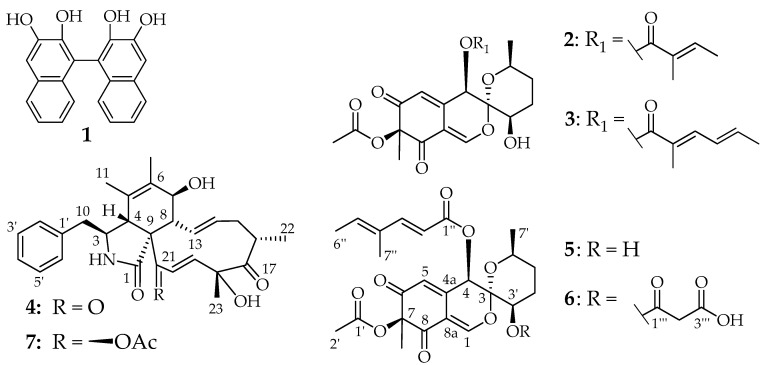
Chemical structures of secondary metabolites of the *Hypoxylon fuscum* complex: BNT (**1**), daldinin C (**2**), daldinin E (**3**), pseudofuscochalasin A (**4**), daldinin F (**5**), 3′-malonyl-daldinin F (**6**) and cytochalasin C (**7**).

**Table 1:** Isolation and accession numbers of sequences used in the phylogenetic analyses. Type specimens are labeled with HT (holotype) ET (epitype) and PT (paratype). Isolates/sequences in bold were isolated/sequenced in present study. N/A: not available.

Species	Strain Number	Origin	Status	GenBank Accession Number				References
				ITS	LSU	RPB2	TUB2	
*Annulohypoxylon annulatum*	CBS 140775	Texas	ET	KY610418	KY610418	KY624263	KX376353	[2,23]
*Annulohypoxylon michelianum*	CBS 119993	Spain		KX376320	KY610423	KY624234	KX271239	[2,29]
*Annulohypoxylon moriforme*	CBS 123579	Martinique		KX376321	KY610425	KY624289	KX271261	[2,23]
*Annulohypoxylon nitens*	MFLUCC 12–0823	Thailand		KJ934991	KJ934992	KJ934994	KJ934993	[30]
*Annulohypoxylon stygium*	MUCL 54601	French Guiana		KY610409	KY610475	KY624292	KX271263	[2]
*Annulohypoxylon truncatum*	CBS 140778	Texas	ET	KY610419	KY610419	KY624277	KX376352	[2,23]
*Biscogniauxia nummularia*	MUCL 51395	France	ET	KY610382	KY610427	KY624236	KX271241	[2]
*Daldinia andina*	CBS 114736	Ecuador	HT	AM749918	KY610430	KY624239	KC977259	[2,29,31]
*Daldinia bambusicola*	CBS 122872	Thailand	HT	KY610385	KY610431	KY624241	AY951688	[2,6]
*Daldinia caldariorum*	MUCL 49211	France		AM749934	KY610433	KY624242	KC977282	[2,29,31]
*Daldinia childiae*	CBS 122881	France	HT	KU683757	MH874773	KU684290	KU684129	[32,33]
*Daldinia concentrica*	CBS 113277	Germany		AY616683	KY610434	KY624243	KC977274	[2,17,29]
*Daldinia dennisii*	CBS 114741	Australia	HT	JX658477	KY610435	KY624244	KC977262	[2,29,34]
*Daldinia eschscholtzii*	MUCL 45435	Benin		JX658484	KY610437	KY624246	KC977266	[2,29,34]
*Daldinia loculatoides*	CBS 113279	UK	ET	AF176982	KY610438	KY624247	KX271246	[2]
*Daldinia macaronesica*	CBS 113040	Spain	PT	KY610398	KY610477	KY624294	KX271266	[2]
*Daldinia petriniae*	MUCL 49214	Austria	ET	AM749937	KY610439	KY624248	KC977261	[2,29,31]
*Daldinia placentiformis*	MUCL 47603	Mexico		AM749921	KY610440	KY624249	KC977278	[2,29,31]
*Daldinia pyrenaica*	MUCL 53969	France		KY610413	KY610413	KY624274	KY624312	[2]
*Daldinia steglichii*	MUCL 43512	Papua New Guinea	PT	KY610399	KY610479	KY624250	KX271269	[2]
*Daldinia theissenii*	CBS 113044	Argentina	PT	KY610388	KY610441	KY624251	KX271247	[2]
*Daldinia vernicosa*	CBS 119316	Germany	ET	KY610395	KY610442	KY624252	KC977260	[2,29]
*Entonaema liquescens*	ATCC 46302	USA		KY610389	KY610443	KY624253	KX271248	[2]
*Graphostroma platystomum*	CBS 270.87	France		JX658535	DQ836906	KY624296	HG934108	[2,34,35,36]
*Hypomontagnella barbarensis*	STMA 14081	Argentina	HT	MK131720	MK131718	MK135891	MK135893	[7]
*Hypomontagnella monticulosa*	MUCL 54604	French Guiana	ET	KY610404	KY610487	KY624305	KX271273	[2]
*Hypomontagnella submonticulosa*	CBS 115280	France		KC968923	KY610457	KY624226	KC977267	[2,29]
*Hypoxylon carneum*	MUCL 54177	France		KY610400	KY610480	KY624297	KX271270	[2]
*Hypoxylon cercidicola*	CBS 119009	France		KC968908	KY610444	KY624254	KC977263	[2,29]
*Hypoxylon crocopeplum*	CBS 119004	France		KC968907	KY610445	KY624255	KC977268	[2,29]
***Hypoxylon eurasiaticum***	MUCL 57720	Iran	HT	**MW367851**	not obtained	**MW373852**	**MW373861**	This study
***Hypoxylon eurasiaticum***	MUCL 57721	Iran		**MW367852**	not obtained	**MW373853**	**MW373862**	This study
***Hypoxylon eurasiaticum***	MUCL 57722	Iran		**MW367853**	not obtained	**MW373854**	**MW373863**	This study
***Hypoxylon eurasiaticum***	MUCL 57723	Iran		**MW367854**	not obtained	**MW373855**	**MW373864**	This study
***Hypoxylon eurasiaticum***	DSM 112037	Poland		**MW367855**	not obtained	**MW373856**	**MW373865**	This study
*Hypoxylon fendleri*	MUCL 54792	French Guiana		KF234421	KY610481	KY624298	KF300547	[2,29]
*Hypoxylon fragiforme*	MUCL 51264	Germany	ET	KC477229	KM186295	KM186296	KX271282	[2,30,37]
*Hypoxylon fuscum sensu stricto*	CBS 113049	France	ET	KY610401	KY610482	KY624299	KX271271	[2]
***Hypoxylon fuscum s. str.***	DSM 112039	Austria		**MW367856**	**MW367847**	**MW373857**	**MW373866**	This study
*Hypoxylon griseobrunneum*	CBS 331.73	India	HT	KY610402	KY610483	KY624300	KC977303	[2,29]
*Hypoxylon guilanense*	MUCL 57726	Iran	HT	MT214997	MT214992	MT212235	MT212239	[21]
*Hypoxylon haematostroma*	MUCL 53301	Martinique	ET	KC968911	KY610484	KY624301	KC977291	[2,29]
*Hypoxylon howeanum*	MUCL 47599	Germany		AM749928	KY610448	KY624258	KC977277	[2,29,31]
*Hypoxylon hypomiltum*	MUCL 51845	Guadeloupe		KY610403	KY610449	KY624302	KX271249	[2]
*Hypoxylon invadens*	MUCL 51475	France	HT	MT809133	MT809132	MT813037	MT813038	[38]
*Hypoxylon investiens*	CBS 118183	Malaysia		KC968925	KY610450	KY624259	KC977270	[2,29]
*Hypoxylon lateripigmentum*	MUCL 53304	Martinique	HT	KC968933	KY610486	KY624304	KC977290	[2,29]
*Hypoxylon lenormandii*	CBS 119003	Ecuador		KC968943	KY610452	KY624261	KC977273	[2,29]
*Hypoxylon ochraceum*	MUCL 54625	Martinique	ET	KC968937	N/A	KY624271	KC977300	[2,29]
*Hypoxylon lienhwacheense*	MFLUCC 14-1231	Thailand		KU604558	MK287550	MK287563	KU159522	[39,40]
*Hypoxylon musceum*	MUCL 53765	Guadeloupe		KC968926	KY610488	KY624306	KC977280	[2,29]
*Hypoxylon olivaceopigmentum*	DSM 107924	USA	HT	MK287530	MK287542	MK287555	MK287568	[39]
*Hypoxylon papillatum*	ATCC 58729	USA	HT	KC968919	KY610454	KY624223	KC977258	[2,29]
*Hypoxylon perforatum*	CBS 115281	France		KY610391	KY610455	KY624224	KX271250	[2]
*Hypoxylon petriniae*	CBS 114746	France	HT	KY610405	KY610491	KY624279	KX271274	[2,23]
*Hypoxylon pilgerianum*	STMA 13455	Martinique		KY610412	KY610412	KY624308	KY624315	[2]
*Hypoxylon porphyreum*	CBS 119022	France		KC968921	KY610456	KY624225	KC977264	[2,29]
***Hypoxylon pseudofuscum***	DSM 112038	Germany	HT	**MW367857**	**MW367848**	**MW373858**	**MW373867**	This study
***Hypoxylon pseudofuscum***	DSM 112035	Germany		**MW367858**	**MW367849**	**MW373859**	**MW373868**	This study
*Hypoxylon pseudofuscum*	DSM 112036	Germany		MW367859	MW367850	MW373860	MW373869	This study
*Hypoxylon pulicicidum*	CBS 122622	Martinique	HT	JX183075	KY610492	KY624280	JX183072	[2,41]
*Hypoxylon rickii*	MUCL 53309	Martinique	ET	KC968932	KY610416	KY624281	KC977288	[2,29]
*Hypoxylon rubiginosum*	MUCL 52887	Germany	ET	KC477232	KY610469	KY624266	KY624311	[2,29]
*Hypoxylon samuelsii*	MUCL 51843	Guadeloupe	ET	KC968916	KY610466	KY624269	KC977286	[2,29]
*Hypoxylon texense*	DSM 107933	USA	HT	MK287536	MK287548	MK287561	MK287574	[39]
*Hypoxylon ticinense*	CBS 115271	France		JQ009317	KY610471	KY624272	AY951757	[2,6]
*Hypoxylon trugodes*	MUCL 54794	Sri Lanka	ET	KF234422	KY610493	KY624282	KF300548	[2,29]
*Hypoxylon vogesiacum*	CBS 115273	France		KC968920	KY610417	KY624283	KX271275	[2,23,29]
*Jackrogersella cohaerens*	CBS 119126	Germany		KY610396	KY610497	KY624270	KY624314	[2]
*Jackrogersella minutella*	CBS 119015	Portugal		KY610381	KY610424	KY624235	KX271240	[2,23]
*Jackrogersella multiformis*	CBS 119016	Germany	ET	KC477234	KY610473	KY624290	KX271262	[2,23,29]
*Pyrenopolyporus hunteri*	MUCL 52673	Ivory Coast	ET	KY610421	KY610472	KY624309	KU159530	[2,23]
*Pyrenopolyporus laminosus*	MUCL 53305	Martinique	HT	KC968934	KY610485	KY624303	KC977292	[2,29]
*Pyrenopolyporus nicaraguensis*	CBS 117739	Burkina_Faso		AM749922	KY610489	KY624307	KC977272	[2,29,31]
*Rhopalostroma angolense*	CBS 126414	Ivory Coast		KY610420	KY610459	KY624228	KX271277	[2]
*Ruwenzoria pseudoannulata*	MUCL 51394	D. R. Congo	HT	KY610406	KY610494	KY624286	KX271278	[2]
*Thamnomyces dendroidea*	CBS 123578	French Guiana	HT	FN428831	KY610467	KY624232	KY624313	[2,42]
*Xylaria arbuscula*	CBS 126415	Germany		KY610394	KY610463	KY624287	KX271257	[2,43]
*Xylaria hypoxylon*	CBS 122620	Sweden	ET	KY610407	KY610495	KY624231	KX271279	[2,44]

**Table 2:** Diagnostic characters of the *Hypoxylon fuscum* complex.

Taxon	Designation No/(Status)	Ascospores (µm)	Mean (µm)	Host	Known Distribution	KOH-Extractable Pigments	Secondary Metabolites
*Hypoxylon fuscum sensu stricto*	Ww3723/Epitype	12.5–15.5 × 5–7	13.2 × 5.8	*Corylus avellana*	Europe	Amber (47) to Honey (64)	**1,5**
*Hypoxylon fuscum sensu stricto*	WU 43621	13–15.8× 4.8–6	14.4 × 5.4	*Corylus avellana*	Austria	Amber (47) to Honey (64)	**1,5**
*Hypoxylon eurasiaticum*	GUM 1597 (H)	10–12.5 × 4.5–6	11.25 × 5.25	*Quercus castaneifolia*	Iran	Amber (47) to Orange (7)	**1,5,6**
*Hypoxylon eurasiaticum*	KR-M-0005886	9–12 × 3.8–5	10.5 × 4.4	cf. *Betula*	Poland	Isabelline (65), Olivaceous (47) or Hazel (88)	**1,5,6**
*Hypoxylon pseudofuscum*	KR-M-0005879 (H)	12–16 × 4.8–7.3	14 × 6	*Alnus glutinosa*	Germany	Isabelline (65), or Hazel (88)	**1,4,5,6**
*Hypoxylon pseudofuscum*	GUM 987	11–15 × 4.5–6.5	13 × 5.5	*Alnus* sp.	Iran	Amber (47) to Oramge (7)	**1,4,5,6**
*Hypoxylon pseudofuscum*	KR-M-0005877/ KR-M-0005876	11–15 × 5.5–6.5	13 × 6	*Salix* sp.	Germany	Isabelline (65), Olivaceous (47) or Hazel (88)	**1,4,5,6**

**Table 3:** Summary of chemotaxonomic results on selected specimens. **1**: BNT; **4**: Pseudofuscochalasin A; **5:** Daldinin F **6**: 3′malonyl-Daldinin F;. **UC**: Unidentified compound.

Species	Specimen	Plant Host	Origin	1	4	5	6	UC 1	UC 2	UC 3
*Hypoxylon fuscum sensu stricto*	WU 43621	*Corylus avellana*	Austria	+	−	+	−	−	+	+
*Hypoxylon eurasiaticum*	GUM 1597 (H)	*Quercus castaneifolia*	Iran	+	−	+	+	−	+	+
*Hypoxylon eurasiaticum*	GUM 1598	*Quercus castaneifolia*	Iran	+	−	+	+	−	+	+
*Hypoxylon eurasiaticum*	GUM 1600	*Quercus castaneifolia*	Iran	+	−	+	+	−	+	+
*Hypoxylon eurasiaticum*	GUM 988	*Quercus castaneifolia*	Iran	+	−	+	+	−	+	+
*Hypoxylon eurasiaticum*	KR-M-0005886	cf*. Betula*	Poland	+	−	+	+	−	+	+
*Hypoxylon pseudofuscum*	KR-M-0005879 (H)	*Alnus glutinosa*	Germany	+	+	+	+	+	+	+
*Hypoxylon pseudofuscum*	GUM 987	*Alnus* sp.	Iran	+	+	+	+	+	+	+
*Hypoxylon pseudofuscum*	KR-M-0005877	*Salix* sp.	Germany	+	+	+	+	+	+	+
*Hypoxylon pseudofuscum*	KR-M-0005876	*Salix* sp.	Germany	+	+	+	+	+	+	+

## Supplementary Materials

The following are available online at https://www.mdpi.com/2309-608X/7/2/131/s1, Figure S1: HRESIMS data of pseudofuscochalasin A (**4**); Figure S2: ^1^H NMR spectrum (500 MHz, chloroform-d) of pseudofuscochalasin A (**4**); Figure S3: ^13^C NMR spectrum (125 MHz, chloroform-*d*) of pseudofuscochalasin A (**4**); Figure S4: COSY NMR spectrum (500 MHz, chloroform-*d*) of pseudofuscochalasin A (**4**); Figure S5: ROESY NMR spectrum (500 MHz, chloroform-*d*) of pseudofuscochalasin A (**4**); Figure S6: HSQC NMR spectrum (500 MHz, chloroform-*d*) of pseudofuscochalasin A (**4**); Figure S7: HMBC NMR spectrum (500 MHz, chloroform-*d*) of pseudofuscochalasin A (**4**); Figure S8: HRESIMS data of daldinin F (**5**); Figure S9: ^1^H NMR spectrum (500 MHz, chloroform-*d*) of daldinin F (5); Figure S10: ^13^C NMR spectrum (125 MHz, chloroform-*d*) of daldinin F (**5)**; Figure S11:. HRESIMS data of 3′-malonyl-daldinin F (**6)**; Figure S12^: 1^H NMR spectrum (700 MHz, acetone-*d*_6_) of 3-malonyl daldinin F (**6**); Figure S13: ^13^C NMR spectrum (175 MHz, aceton-*d*_6_) of 3-malonyl daldinin F (**6)**; Figure S14: COSY NMR spectrum (700 MHz, acetone-*d*_6_) of 3-malonyl daldinin F (**6**); Figure S15: ROESY NMR spectrum (700 MHz, aceton-*d*_6_) 3-malonyl daldinin F (**6**); Figure S16: HSQC NMR spectrum (700 MHz, acetone-*d*_6_) 3-malonyl daldinin F (**6**); Figure S17 HMBC NMR spectrum (700 MHz, acetone-*d*_6_) of 3-malonyl daldinin F (**6**).
