# Supplementary material for: Resolution of the Hypoxylon fuscum Complex (Hypoxylaceae, Xylariales) and Discovery and Biological Characterization of Two of Its Prominent Secondary Metabolites
Source: J Fungi (Basel). 2021 Feb 11;7(2):131. doi: 10.3390/jof7020131 (PMC7916920; doi:10.3390/jof7020131)
Supplement: Supplementary file 1 [file jof-07-00131-s001.zip › si-fuscum/SI Fuscumpaper.pdf]

Article

Resolution of the *Hypoxylon fuscum* complex (Hypoxylaceae, Xylariales) and discovery and biological characterization of two of its prominent secondary metabolites

Christopher Lambert[1,2,†,] Mohammad Javad Pourmoghaddam[1, 3,4,†], Marjorie Cedeño–Sanchez[1,2], Frank Surup [1,2], Seyed Akbar Khodaparast[3], Irmgard Krisai-Greilhuber[4], Hermann Voglmayr[4,5], Theresia E.B. Stradal[6,7,*] and Marc Stadler[1,2,7,*]

1 Department for Microbial Drugs, Helmholtz-Centre for Infection Research GmbH, and Institute of Microbiology, Inhoffenstraße 7, 38124 Braunschweig, Germany
2 German Centre for Infection Research (DZIF), Partner Site Hannover-Braunschweig, 38124 Braunschweig, Germany
3 Department of Plant Protection, Faculty of Agricultural Sciences, University of Guilan, Rasht, Iran
4 Department of Botany and Biodiversity Research, University of Vienna, Rennweg 14, 1030, Wien, Austria
5 Institute of Forest Entomology, Forest Pathology and Forest Protection, Department of Forest and Soil Sciences, BOKU-University of Natural Resources and Life Sciences, Franz Schwackhöfer Haus, Peter-Jordan-Straße 82/I, 1190 Vienna, Austria
6 Department for Cell Biology, Helmholtz-Centre for Infection Research GmbH, Inhoffenstraße 7, 38124 Braunschweig, Germany
7 Institute of Microbiology, Technische Universität Braunschweig, Spielmannstraße 7, 38106 Braunschweig
* * Correspondence: marc.stadler@helmholtz-hzi.de; Tel.: +49-531-6181-4240; Fax: +49-531-6181-9499

Supplementary Information

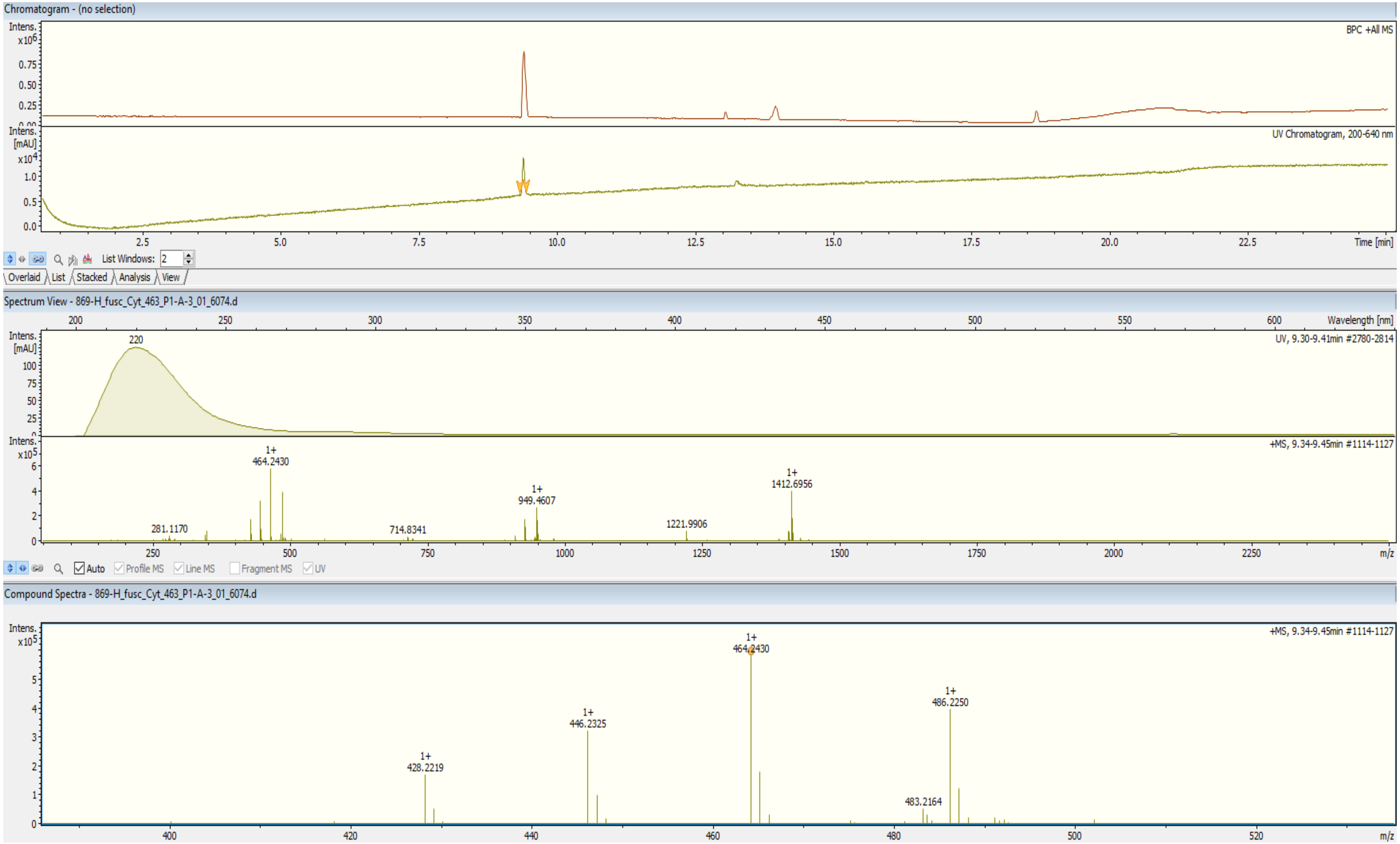

Fig. S1. HRESIMS data of pseudofuscochalasin A (**4**).

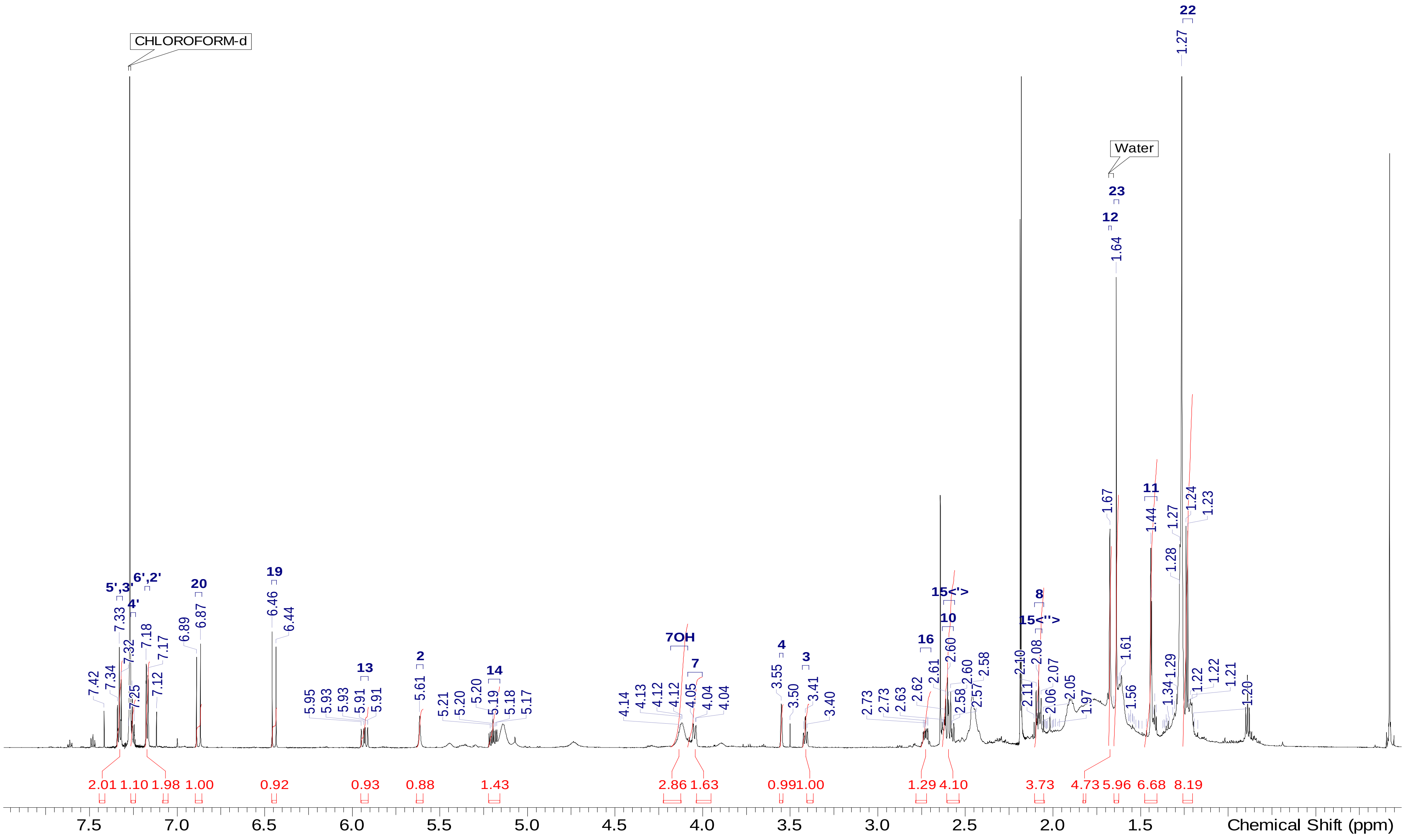

Fig S2. ^{1}H NMR spectrum (500 MHz, chloroform-*d*) of pseudofuscochalasin A (**4**).

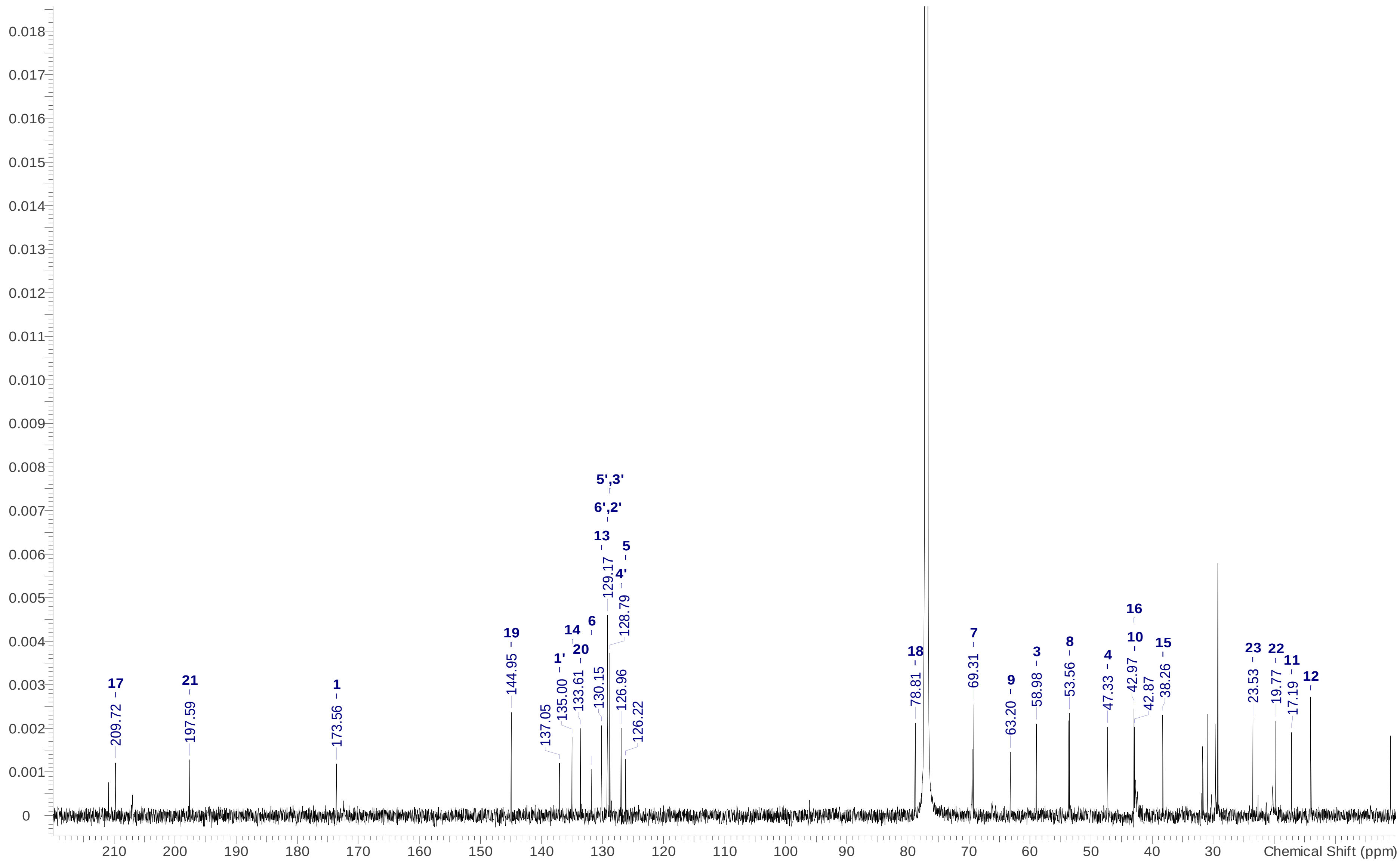

Fig. S3. ^{13}C NMR spectrum (125 MHz, chloroform-*d*) of pseudofuscochalasin A (**4**).

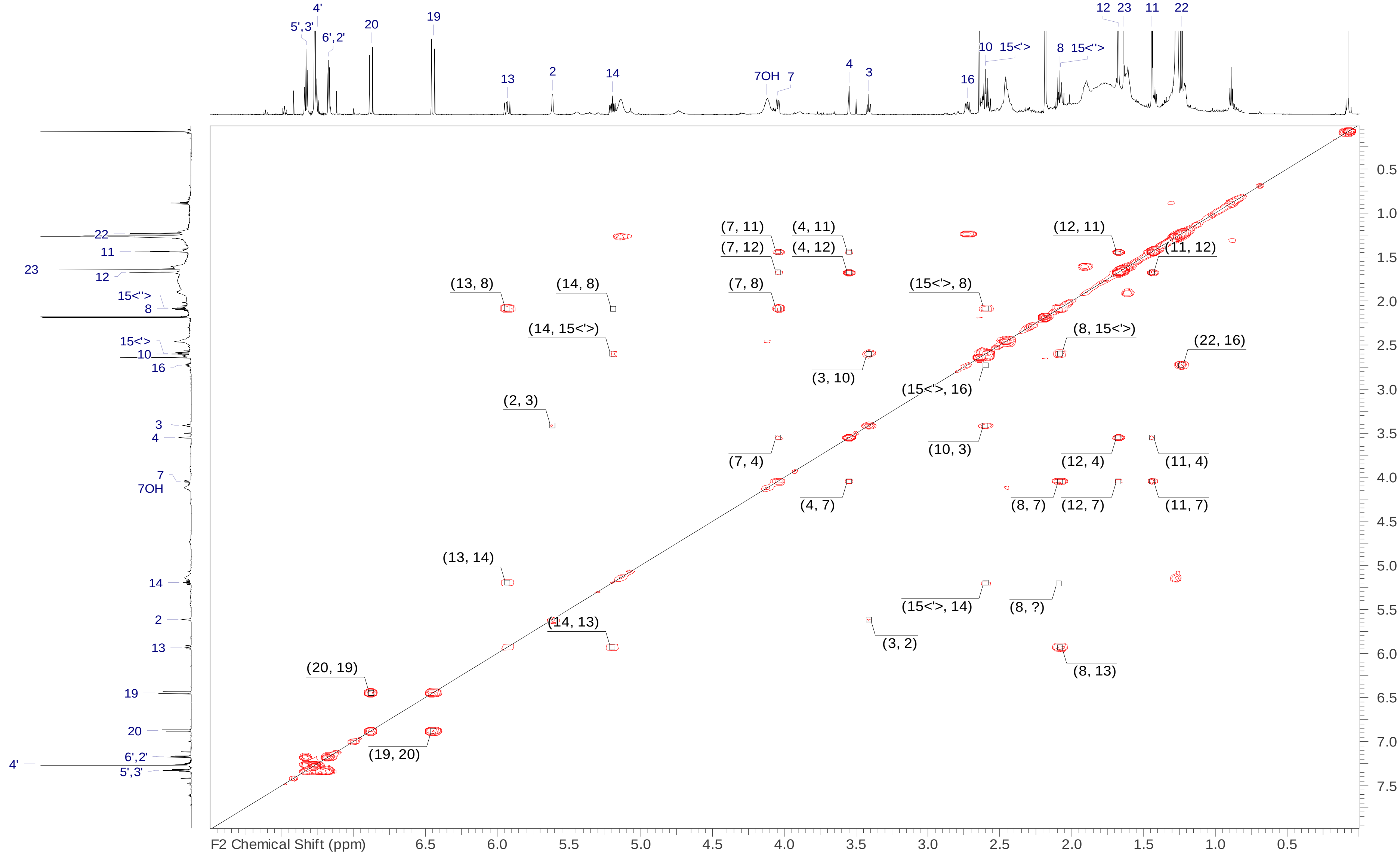

Fig. S4. COSY NMR spectrum (500 MHz, chloroform-*d*) of pseudofuscochalasin A (**4**).

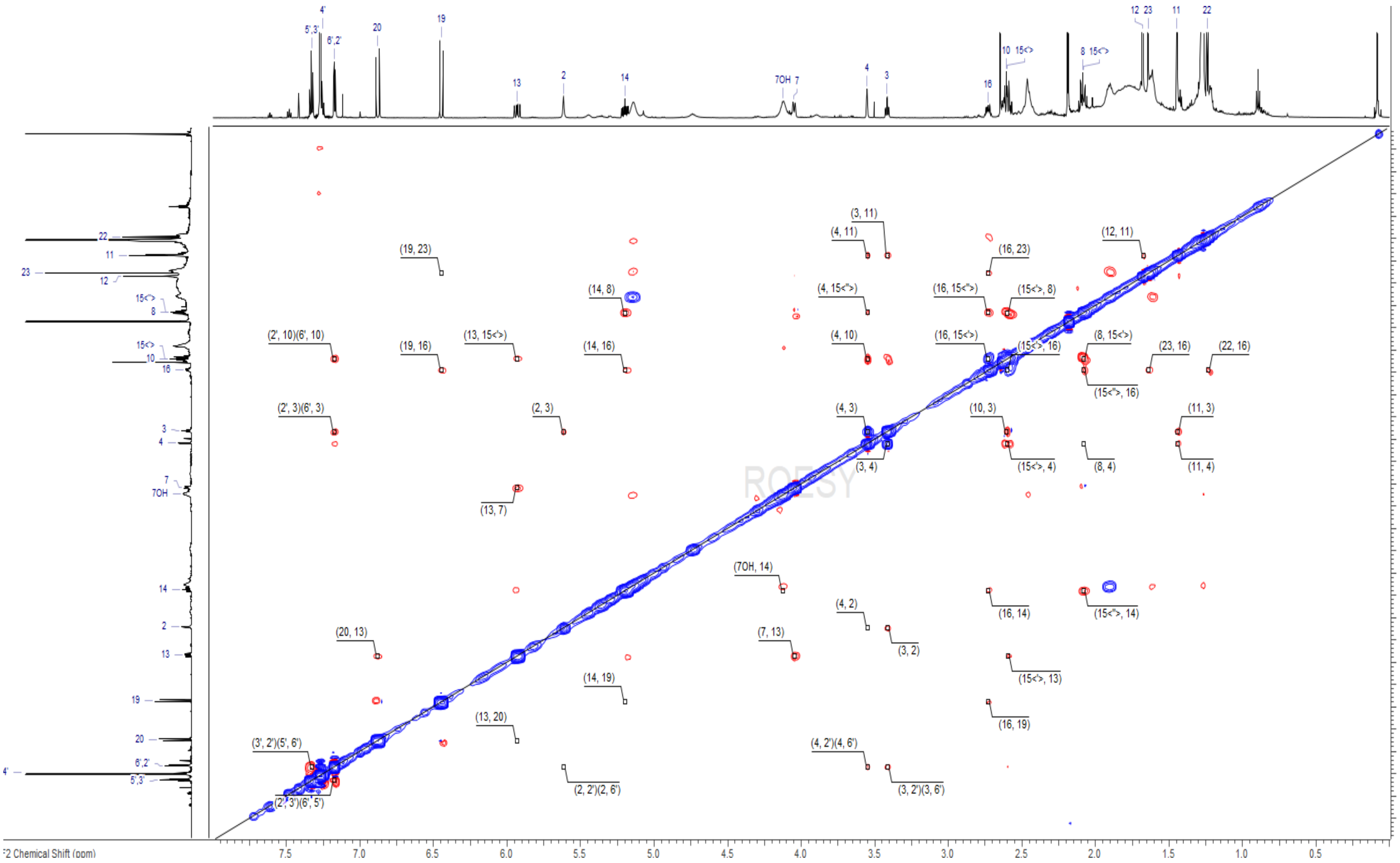

Fig. S5. ROESY NMR spectrum (500 MHz, chloroform-*d*) of pseudofuscochalasin A (**4**).

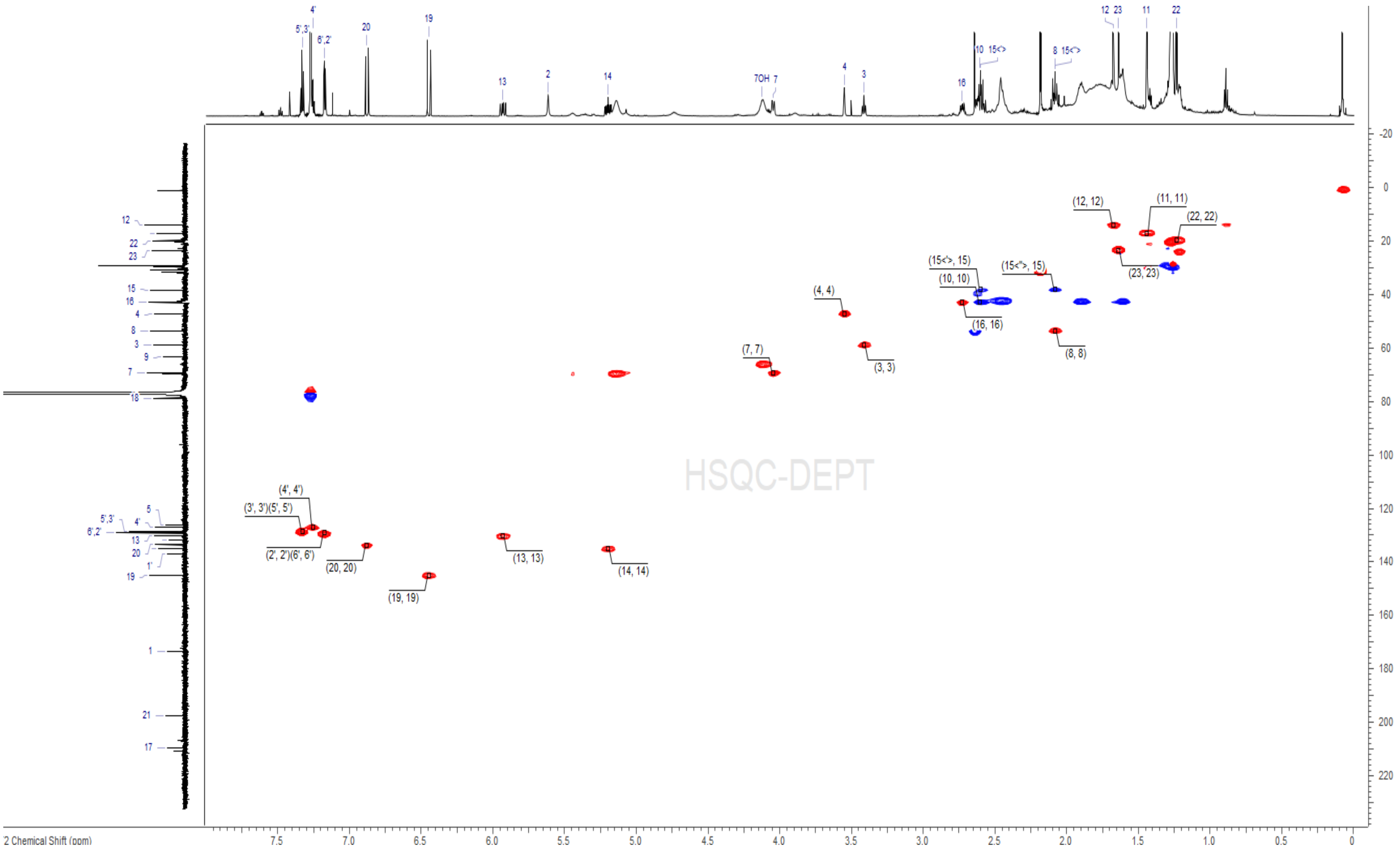

Fig. S6. HSQC NMR spectrum (500 MHz, chloroform-*d*) of pseudofuscochalasin A (**4**).

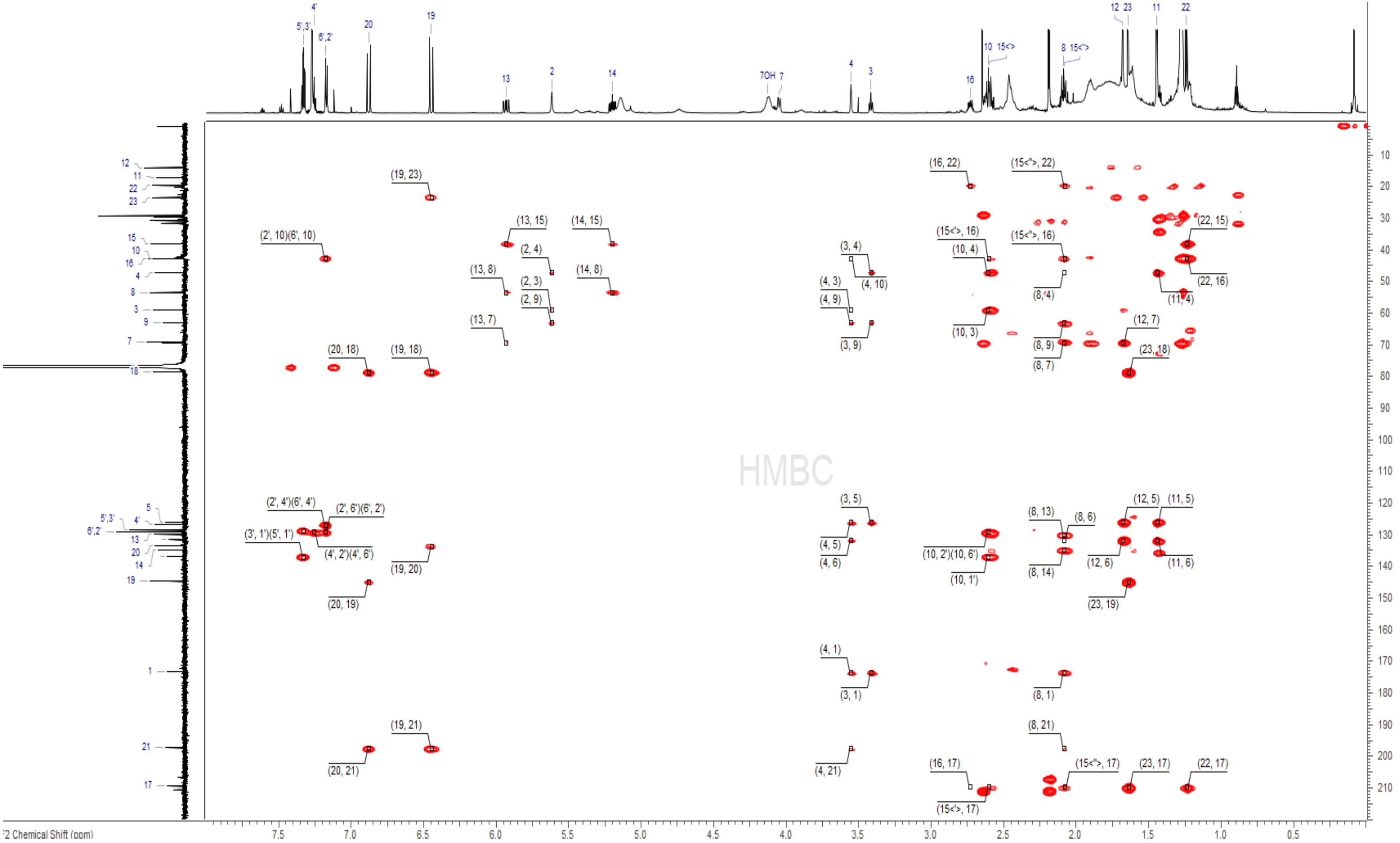

Fig. S7. HMBC NMR spectrum (500 MHz, chloroform-*d*) of pseudofuscochalasin A (**4**).

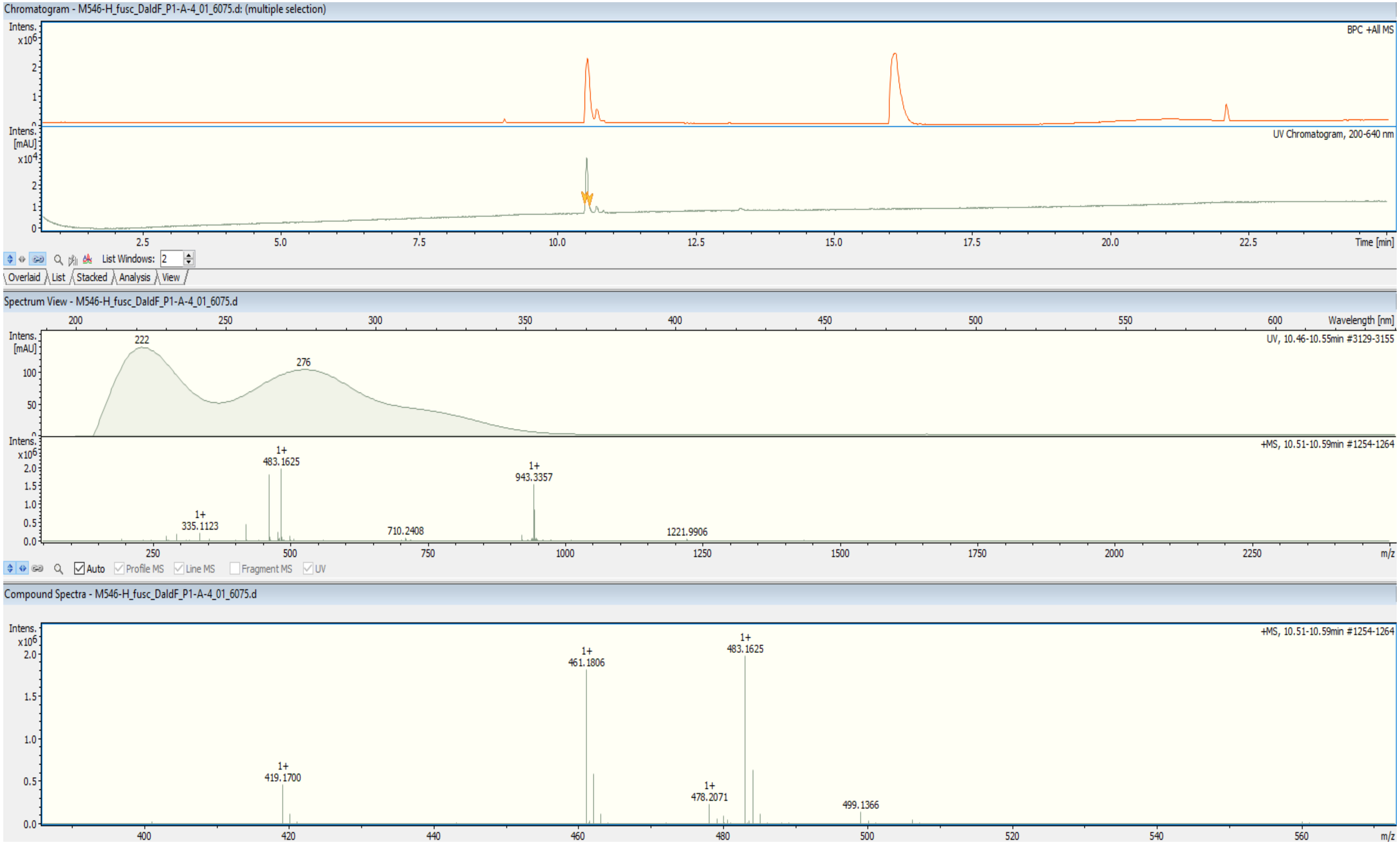

Fig. S8. HRESIMS data of daldinin F (**5**).

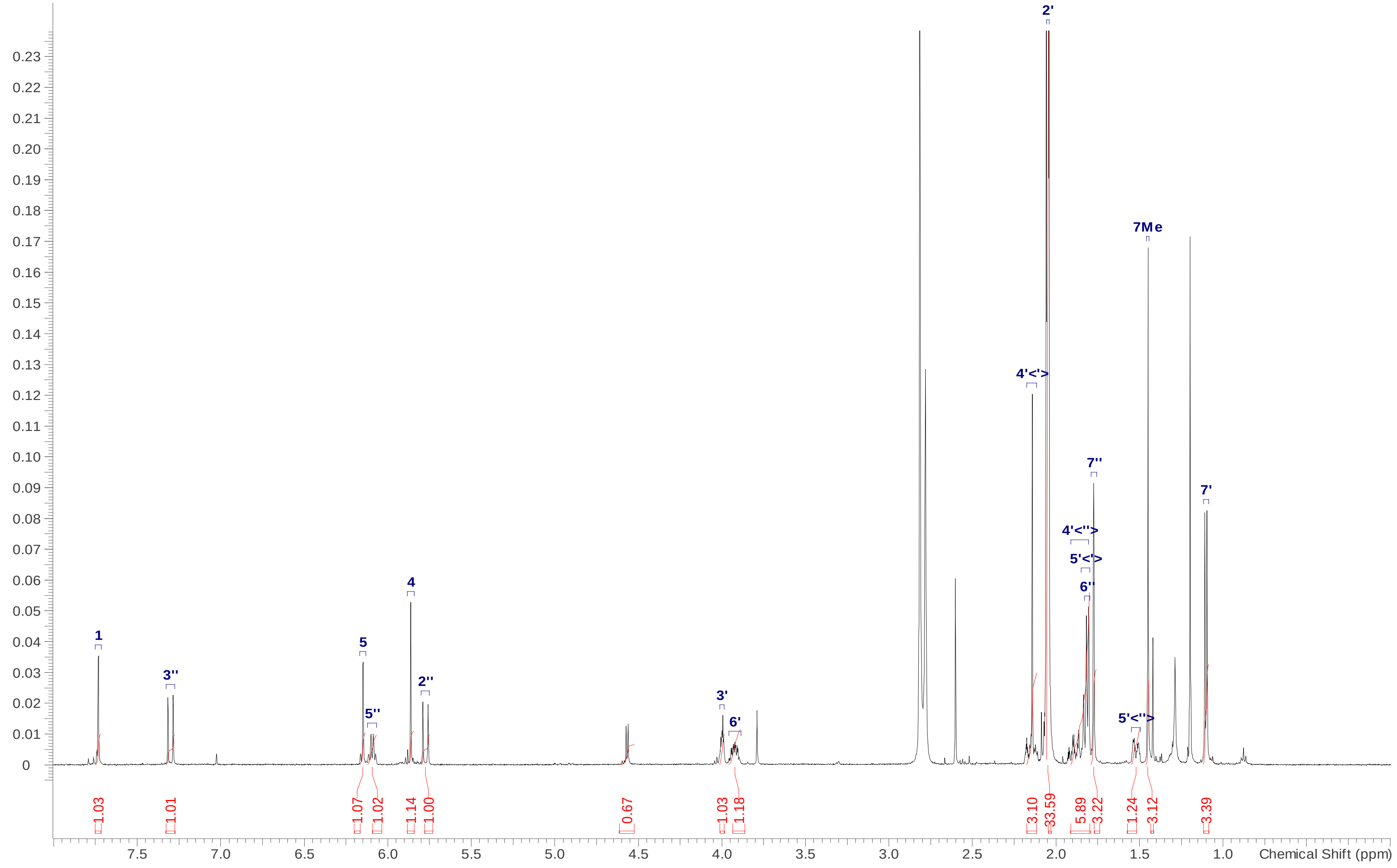

Fig. S9. ^{1}H NMR spectrum (500 MHz, chloroform-*d*) of daldinin F (**5**).

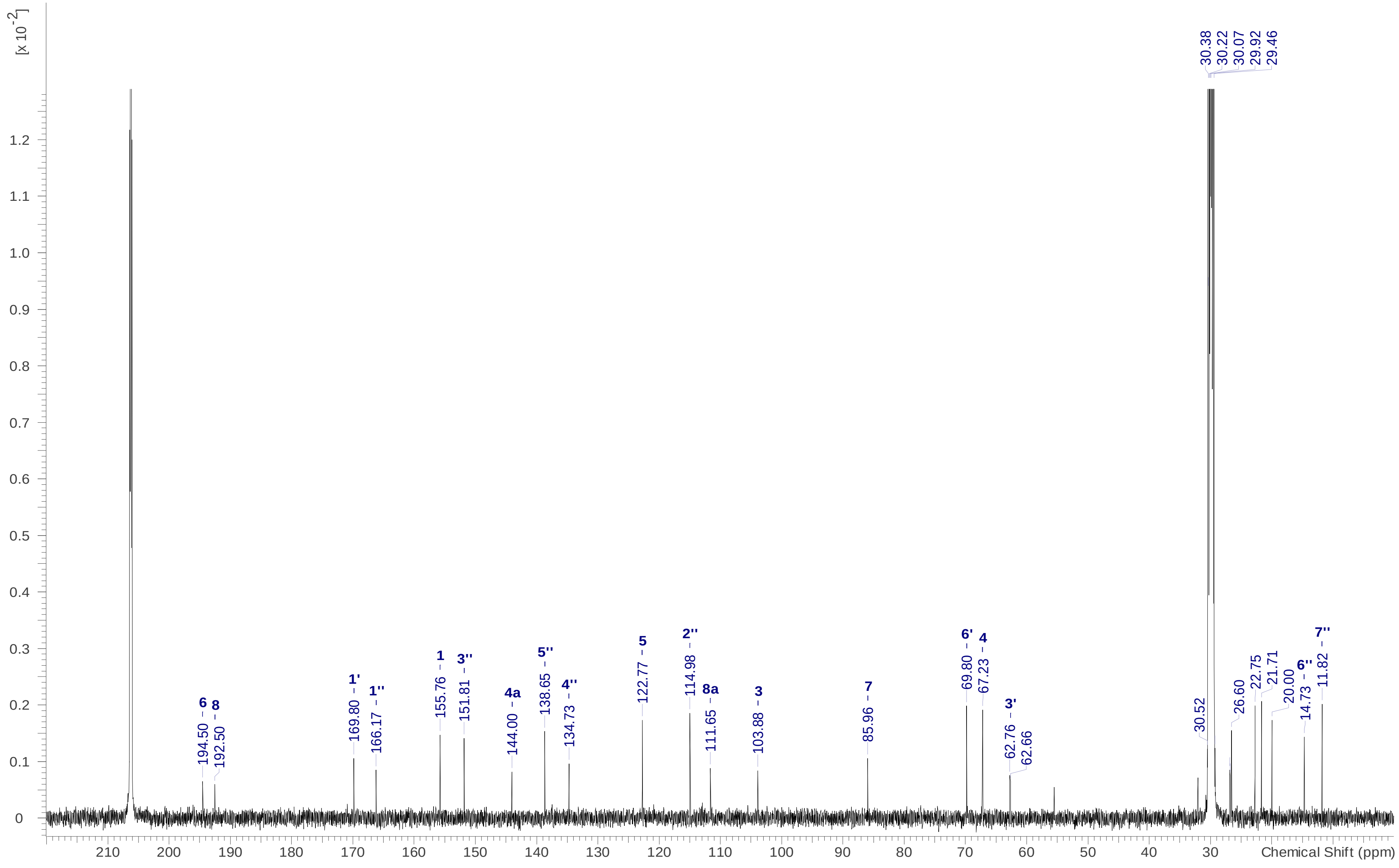

Fig. S10. ^{13}C NMR spectrum (125 MHz, chloroform-*d*) of daldinin F (**5**).

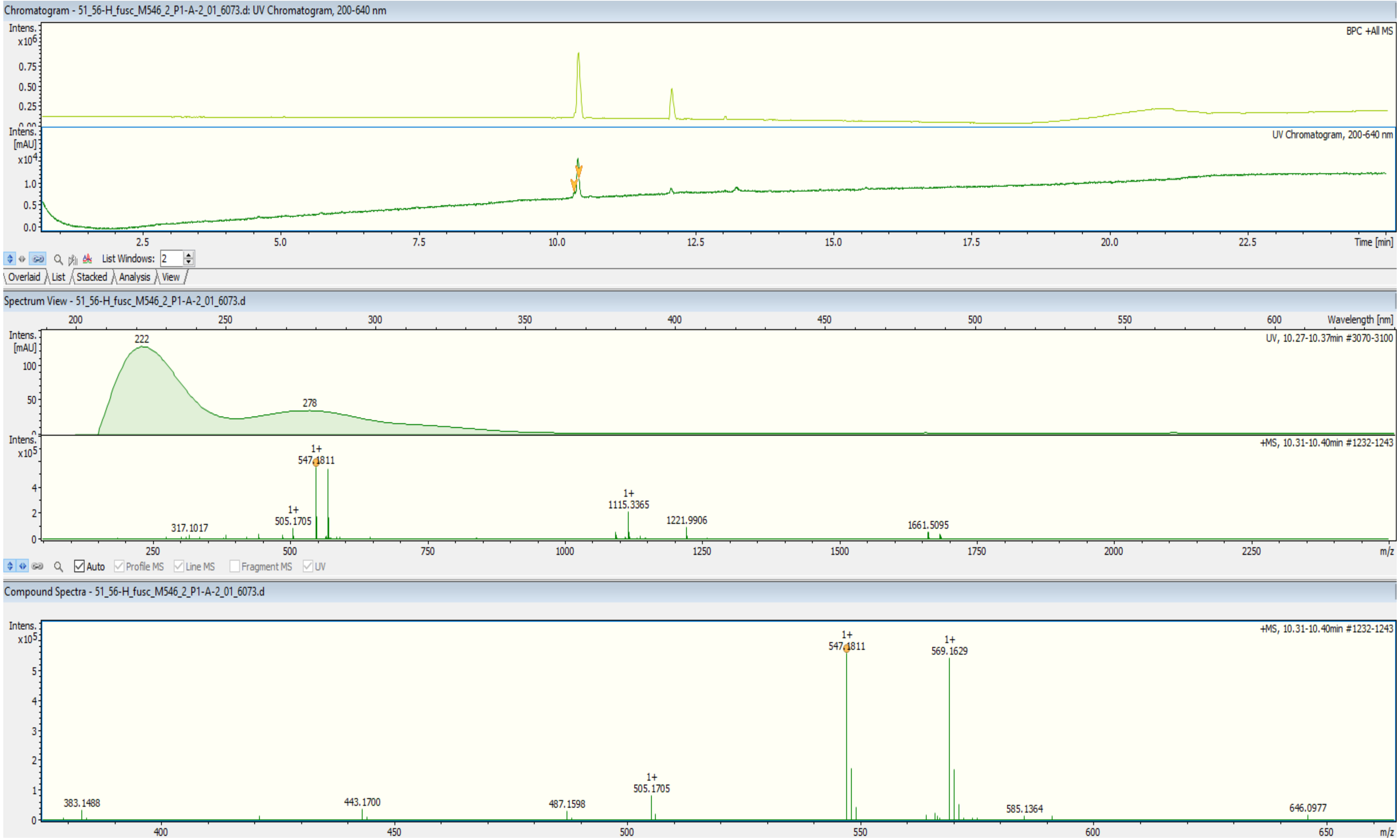

Fig. S11. HRESIMS data of 3′-malonyl-daldinin F (**6**).

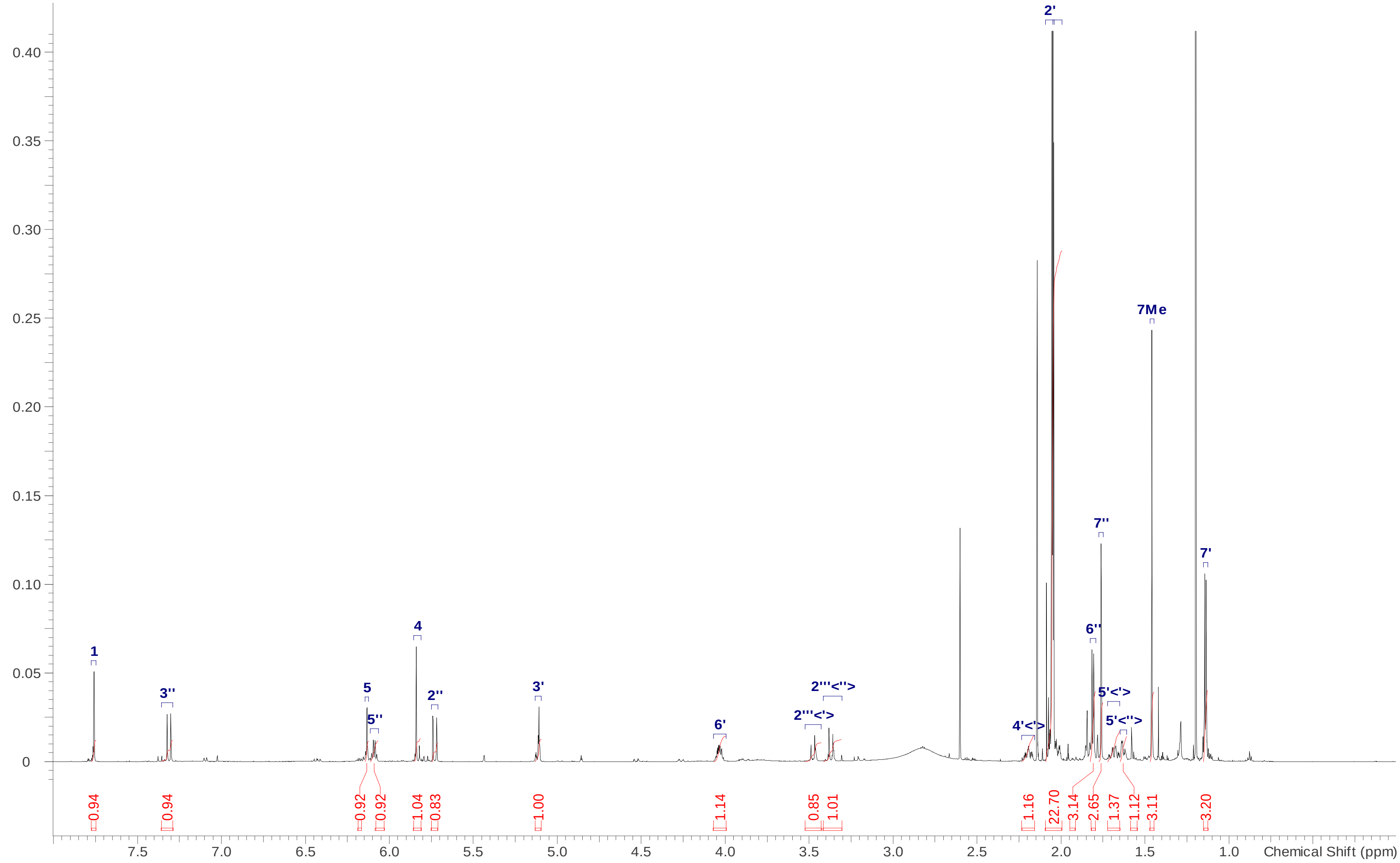

Fig. S12. ^{1}H NMR spectrum (700 MHz, aceton-d_6) of 3-malonyl daldinin F (**6**).

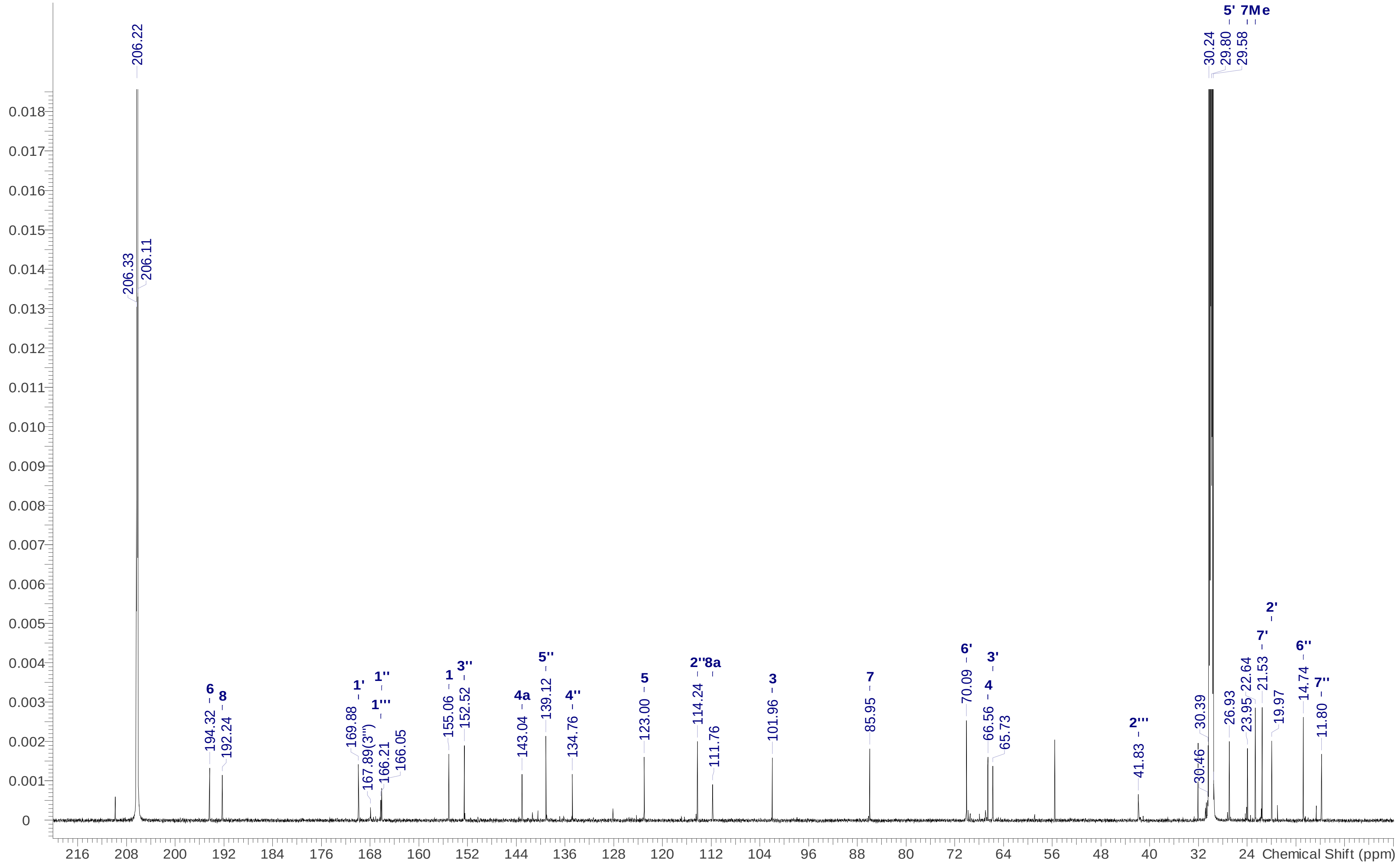

Fig. S13. ^{13}C NMR spectrum (175 MHz, aceton-d_6) of 3-malonyl daldinin F (**6**).

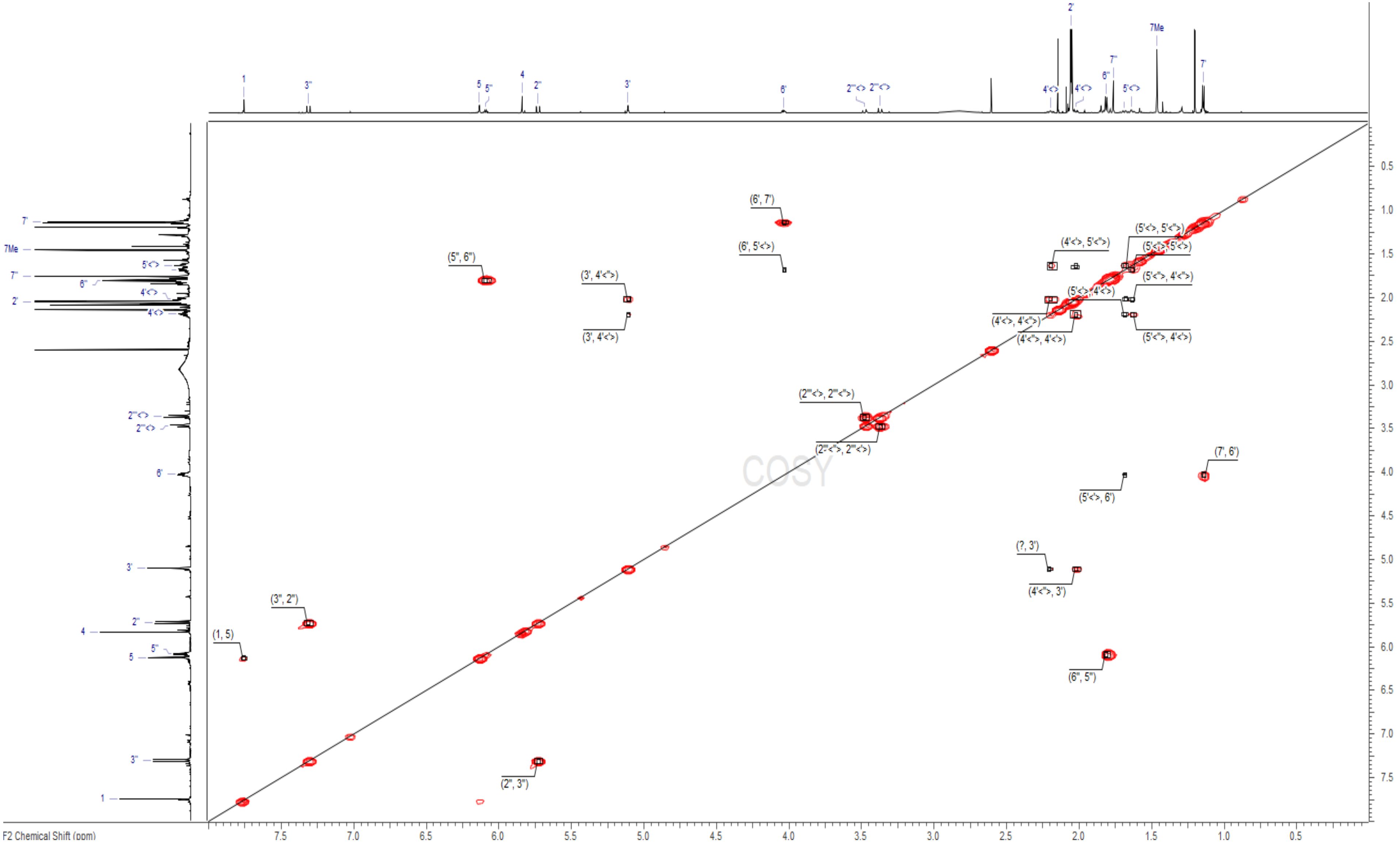

Fig. S14. COSY NMR spectrum (700 MHz, aceton-d_6) 3-malonyl daldinin F (**6**).

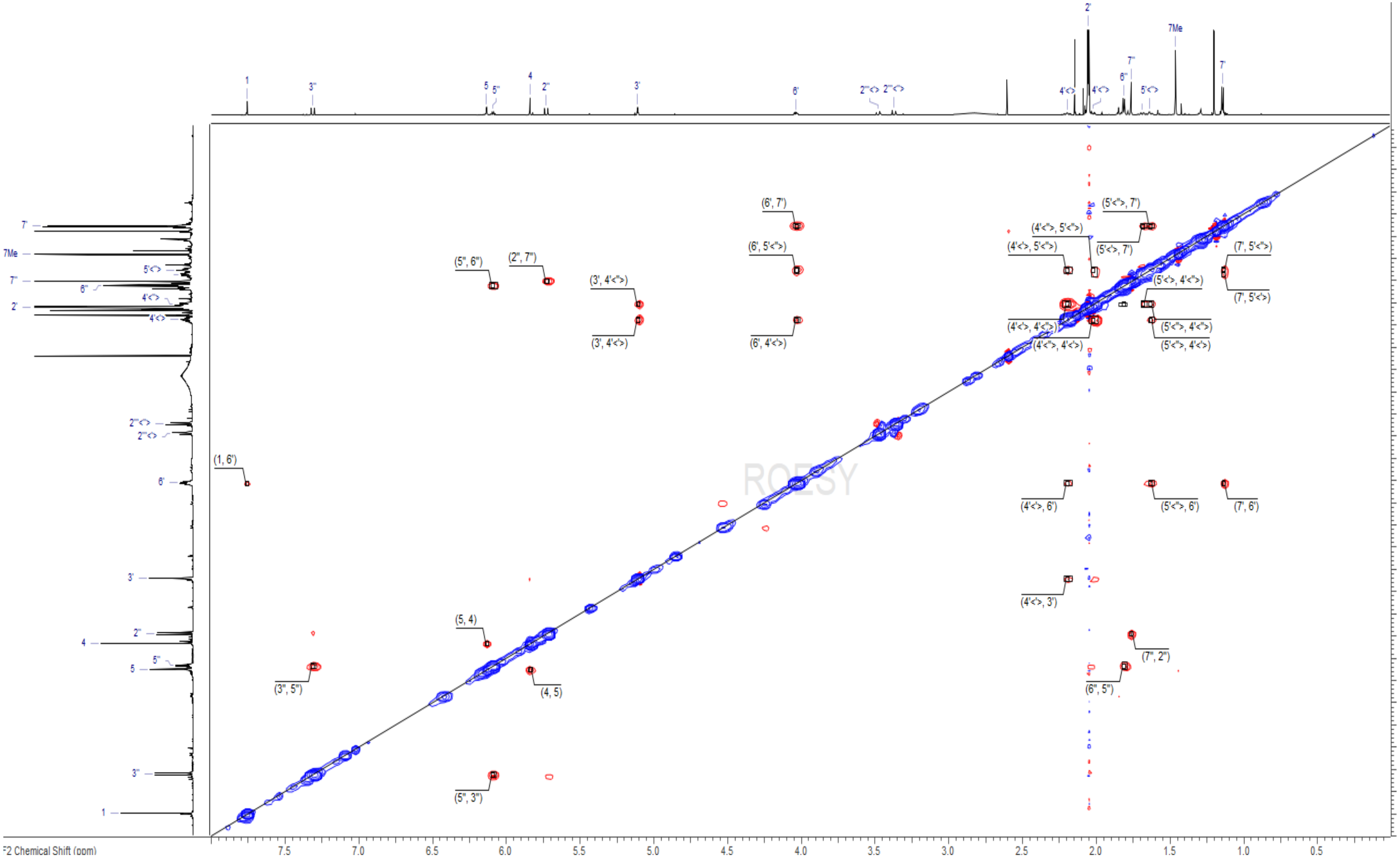

Fig. S15. ROESY NMR spectrum (700 MHz, aceton-d_6) 3-malonyl daldinin F (**6**).

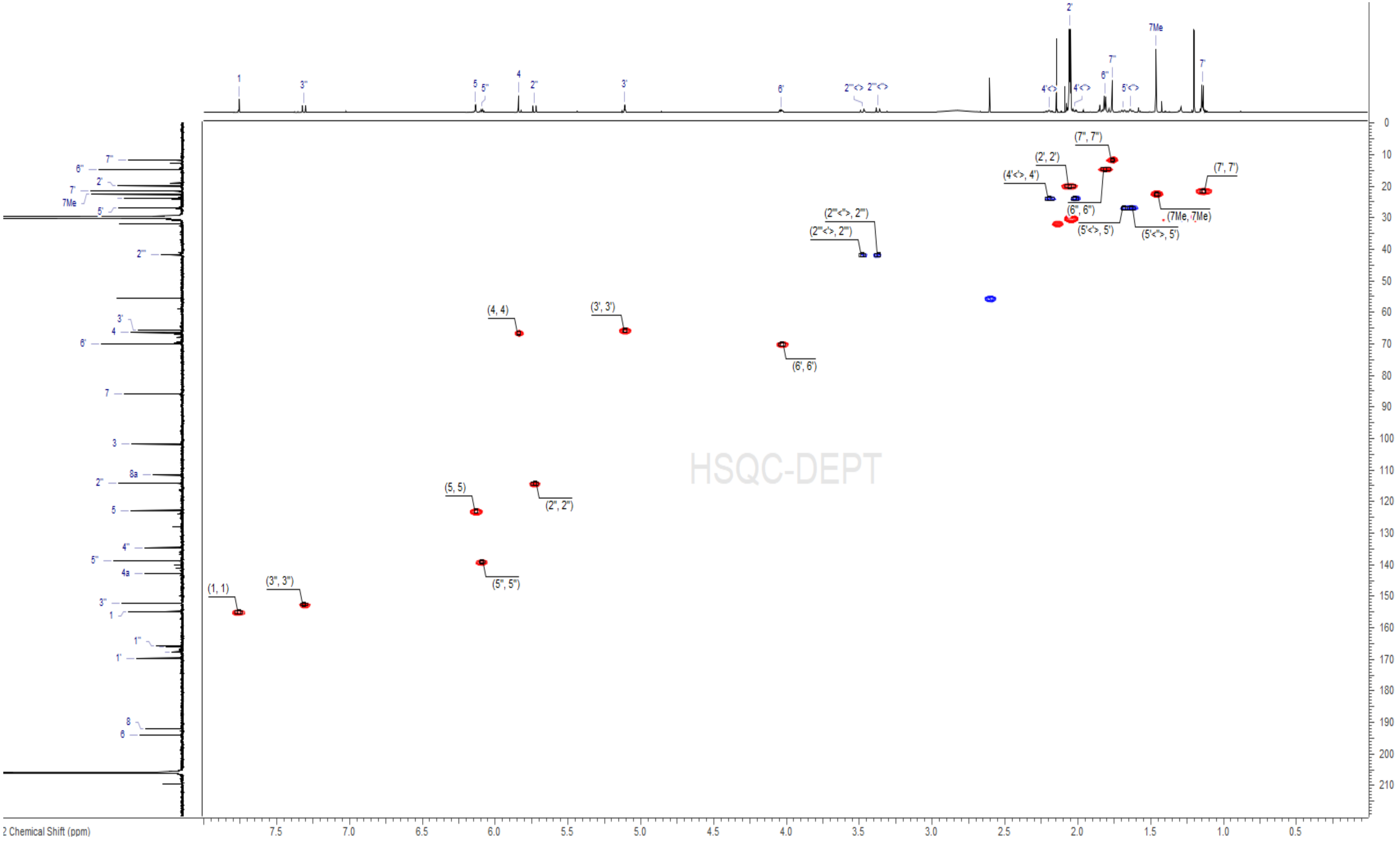

Fig. S16. HSQC NMR spectrum (700 MHz, aceton-d_6) 3-malonyl daldinin F (**6**).

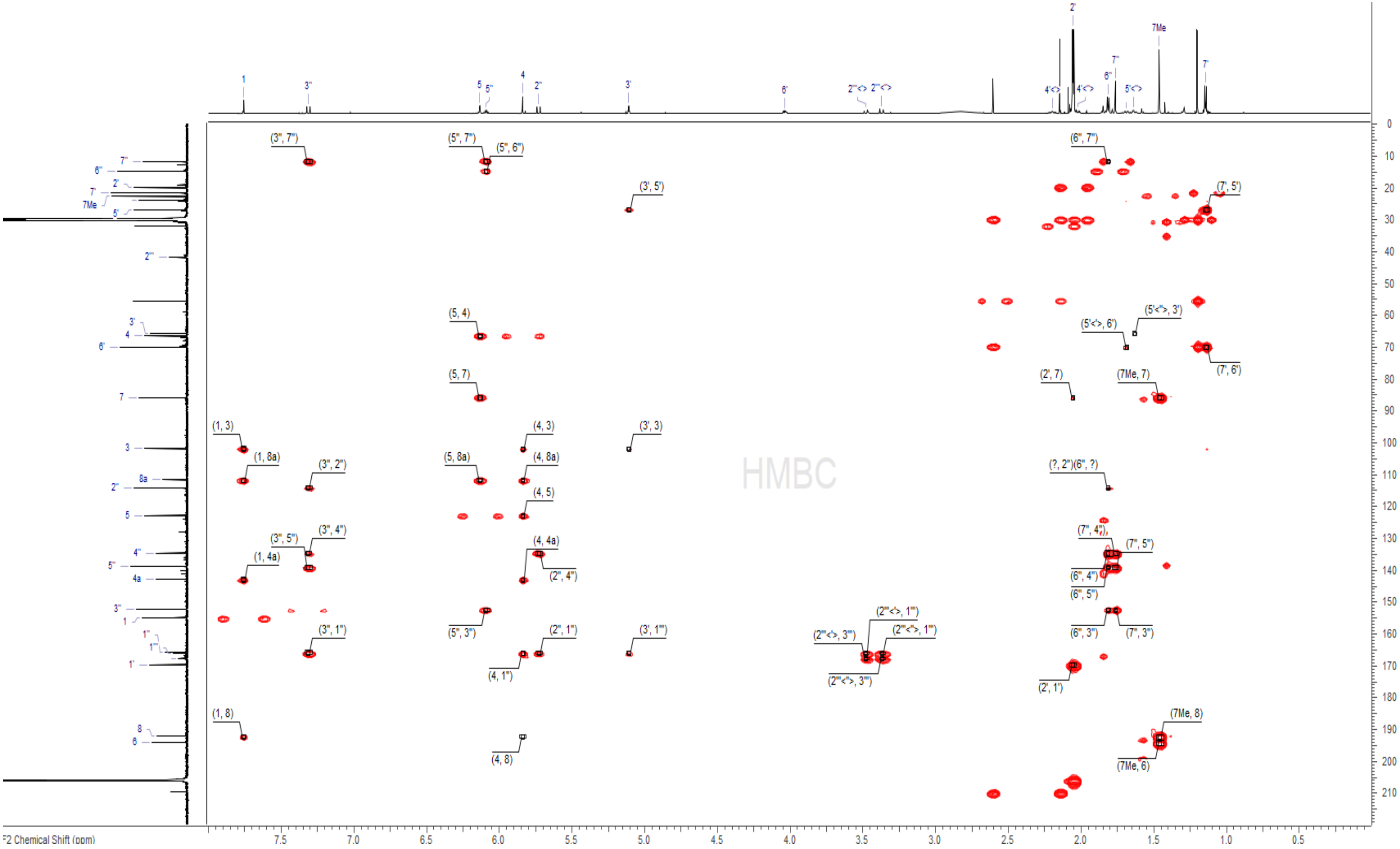

Fig. S17. HMBC NMR spectrum (700 MHz, aceton-d_6) 3-malonyl daldinin F (**6**).
